# Dynamic Privacy-Preserving Anonymous Authentication Scheme for Condition-Matching in Fog-Cloud-Based VANETs

**DOI:** 10.3390/s24061773

**Published:** 2024-03-09

**Authors:** Yonghua Zhan, Weipeng Xie, Rui Shi, Yunhu Huang, Xianghan Zheng

**Affiliations:** 1 College of Computer and Data Science, Fuzhou University, Fuzhou 350108, China; zhanyonghua@126.com (Y.Z.); xianghan.zheng@fzu.edu.cn (X.Z.); 2 Beijing Electronic Science and Technology Institute, Beijing 100070, China; ruishi_mail@126.com; 3 Computer and Data Science, Minjiang University, Fuzhou 350108, China

**Keywords:** VANETs, conditional privacy-preserving, authenticated key agreement, dynamic group, fog-cloud computing

## Abstract

Secure group communication in Vehicle Ad hoc Networks (VANETs) over open channels remains a challenging task. To enable secure group communications with conditional privacy, it is necessary to establish a secure session using Authenticated Key Agreement (AKA). However, existing AKAs suffer from problems such as cross-domain dynamic group session key negotiation and heavy computational burdens on the Trusted Authority (TA) and vehicles. To address these challenges, we propose a dynamic privacy-preserving anonymous authentication scheme for condition matching in fog-cloud-based VANETs. The scheme employs general Elliptic Curve Cryptosystem (ECC) technology and fog-cloud computing methods to decrease computational overhead for On-Board Units (OBUs) and supports multiple TAs for improved service quality and robustness. Furthermore, certificateless technology alleviates TAs of key management burdens. The security analysis indicates that our solution satisfies the communication security and privacy requirements. Experimental simulations verify that our method achieves optimal overall performance with lower computational costs and smaller communication overhead compared to state-of-the-art solutions.

## 1. Introduction

Vehicle Ad hoc Networks (VANETs) play a crucial role in supporting intelligent transportation systems, including data sharing and collaborative processing, within modern urban traffic [1]. The popularity of electric vehicles brings more powerful sensing modules and stronger computation capabilities. VANETs, through the cooperation of On-Board Units (OBUs) and Roadside Units (RSUs), can provide high-speed data communication services between vehicles, guaranteeing the safety of vehicle travel and achieving fully intelligent traffic management. For example, in the event of a traffic accident, relevant vehicles can report the incident to nearby sections through RSUs, guiding nearby vehicles to avoid congested routes [2]. The role of VANETs in intelligent transportation has attracted attention from both industry and academia [3].

Unlike many fixed-terminal networks, VANETs must deal with rapid changes in access and are more prone to attacks like eavesdropping, user tracking, and tampering [4]. In an open VANET, ensuring communication and data security is a key concern. Traditional VANET security protection schemes generally involve a Trusted Authority (TA) that issues certificates to vehicles and RSUs, handles authentication at the access endpoints, and performs critical security algorithms [5]. However, as more vehicles join VANETs, the TA needs to manage a large number of certificates and handle a significant amount of requests, resulting in high computational and storage costs for the TA. Furthermore, due to the TA’s distance from vehicles and RSUs, higher latency is more likely, making it unable to provide real-time services.

To address the drawbacks of a single TA, multi-TA schemes have been introduced into VANETs [6]. In a multi-TA scheme, fog computing TA sub-nodes are deployed on the RSU side, and fog node TAs are managed by a central TA, forming a two-tier TA structure. Vehicles are authenticated and managed by the fog TA nodes on the RSU side, greatly improving the real-time data processing and mitigating the impact caused by DoS attacks. The VANETs architecture with multi-TA composed of fog computing can significantly enhance the service quality of the network [7].

In some previous VANET schemes, vehicles or RSUs directly report the road conditions to the TA [8]. The TA collects real-time data and responds accordingly. However, as the amount of data generated by vehicles rapidly increases, this places a significant computational burden on the TA, and the cost of storing and computing by the TA becomes extremely high. To address this issue, some scholars have combined VANETs with cloud computing [9,10], using cloud computing to store and process data in VANETs, providing VANETs with more elastic computing capabilities. For example, authenticated vehicles have the ability to upload traffic data to the remote cloud, while the TA is only responsible for secure computations such as authentication.

In VANETs, authenticated key agreement is crucial for communication security. A session security key protocol that satisfies session security can be used to construct a communication channel with dynamic members [11]. Furthermore, to provide security and privacy protection for VANETs communication, various scholars have introduced Conditional Privacy-Preserving (CPP) authentication schemes in recent years [12], where the information of vehicles is kept private from all participants except the TA. However, if a vehicle engages in malicious behavior, the TA is able to trace its real identity.

In recent years, many classic solutions have been studied for CPP authentication under VANETs. Lin et al. [13] combined blockchain technology with key derivation algorithms to manage certificates, in order to avoid vehicles storing a large number of keys, but the single TA mode is vulnerable to DoS attacks. Yu et al. [14] used ECC and certificateless aggregate signatures to reduce the computational load of OBUs, but they cannot support dynamic groups. Wang et al. [15] proposed a scheme that achieves conditional privacy protection without using pseudonyms, but it involves operations with bilinear pairs, resulting in high computational costs and unfriendly support for vehicles with low computing power.

### Our Contributions

To summarize, existing schemes still have issues to address. Traditional session AKA solutions lack consideration for cross-domain scenarios and complete group session key negotiation within a single domain. Multi-TA may enhance VANET response speed and capacity but faces challenges due to increasing data volume. Our scheme addresses these issues by introducing a dynamic privacy-preserving anonymous authentication scheme tailored for fog-cloud-based VANETs. It utilizes RSUs as fog computing nodes, incorporates multi-level TAs, and integrates cloud services for storage and computing. Lightweight security algorithms are employed for group session key negotiation to ensure secure VANET communication. The contributions of our proposed scheme include:

- The introduction of an anonymous and dynamic conditional privacy-preserving scheme using basic elliptic curve algorithms and hash functions for low-computing-power OBUs.
- The implementation of certificateless and multi-TA modes to reduce the burden on TAs, improve response speed, and enhance overall VANET robustness. The use of cloud services as an outsourcing platform to expand data processing capabilities and boost VANET performance.
- Security analysis demonstrates satisfaction of VANET security requirements, achieving forward security and resisting attacks. In comprehensive performance, our proposed solution is better than existing similar conditional privacy-preserving schemes in comprehensive performance.

## 2. Related Work

In order to meet the security and privacy protection requirements of vehicle communication in open channels, many researchers have conducted research on conditional privacy protection for VANETs in recent years. These studies are roughly summarized as PKI-based, certificateless, fog-cloud-based, and blockchain-based.

In 2007, Raya et al. [16] introduced the first PKI-based conditional privacy protection authentication system, aiming to enhance the security of vehicle communication through the utilization of anonymous certificates. However, this scheme necessitates the involvement of a Certification Authority (CA) to handle a substantial volume of certificates. Xiong et al. [17] introduced a authentication framework ensuring conditional privacy with support for dynamic members using the Chinese Remainder Theorem. This protocol supports both forward and backward security, but it also faces the problem of certificate management by a single TA. In response to the security update challenges related to Tamper-Proof Device (TPD) keys, Wei et al. [18] introduced a secure updateable conditional privacy protection authentication scheme. This scheme is built upon Shamir’s secret sharing and secure pseudo-random functions to ensure the robustness of the security updates for TPD keys. By using ECC signatures, this scheme improves the transmission speed of messages in emergency situations. To tackle the security challenges associated with heterogeneous vehicle communication in VANETs, Ali et al. [19] introduced an privacy hybrid signcryption scheme with high efficiency. This scheme relies on bilinear pairings to enhance the security of communication among diverse vehicles. They also reduced decryption time by using batch decryption. To address the risk of private key leakage in VANETs, Xiong et al. [20] constructed a dual insurance conditional privacy authentication scheme using ECC. Even if the master key or one of the vehicle keys is leaked, this scheme ensures that valid authentication messages cannot be forged. To provide traceability and credibility of malicious senders, Luo et al. [21] designed a conditional privacy protection authentication protocol using ring signatures and ring signcryption. This protocol provides publicly verifiable algorithms for exposing the real identity of malicious users, but it requires the support of a third-party TA. To address the privacy concerns introduced by the open channels in VANETs, Cai et al. [22] proposed a conditional privacy protection scheme for VANETs using identity-based encryption and ring signatures. They proved the security properties of anonymity, traceability, confidentiality, and unforgeability of the scheme. However, Du et al. [23] pointed out issues in [22] such as the lack of anonymous protection for honest senders. They improved the scheme to achieve sender anonymity and malicious user traceability, as well as resistance to response attacks. Additionally, Zhou et al. [24] proposed a multi-key outsourcing computation scheme for VANETs, which designed an efficient privacy protection information filtering system location-based service. This system eliminates useless encrypted information before authentication, optimizing the computation and communication workload. Based on PKI, the CPP solution can achieve complex functions, but it also faces challenges such as high computational costs for certain cryptographic primitives.

To avoid the burden of managing certificates and keys, many researchers have started to consider certificateless schemes in VANETs. In order to enhance computational speed, Chen et al. [25] proposed a certificateless fully aggregated signature scheme in 2021, which does not increase the length of signatures with the number of vehicles, reducing communication and processing costs. This scheme uses general ECC and hash computations, reducing the computational burden. Ali et al. [26] considered the limited computation power of OBUs and designed a certificate-free conditional privacy authentication scheme without bilinear pairings and mapping to points. They used ECC and ordinary hash functions instead and improved overall efficiency through batch signature verification. Building on the scheme proposed by [26], Zhou et al. [27] proposed a certificateless privacy-preserving authentication scheme which was both secure and lightweight. This solution can resist signature forgery attacks and has fast computational efficiency compared to [26]. Certificateless solutions effectively reduce the pressure of certificate and key management and lower the risk of key leakage. However, TA requires responsibility for participating in the generation of all keys and certificates, which can be a significant burden.

To address the issue of a high workload on a single CA, several fog-cloud-based VANET solutions have been proposed. Goudarzi et al. [28] proposed a fog-based VANET privacy protection authentication protocol, which utilizes Quotient Filter to solve node authentication, and uses fog nodes to reduce system latency and improve system throughput. Zhong et al. [29] proposed a fog computing-based CPP scheme, which supports mobility, low latency, and location awareness through fog computing, and reduces expenses by generating pseudonyms using two hash chains. Navdeti et al. [30] proposed a fog-based VANET privacy protection and secure data sharing scheme. By outsourcing the data to cloud servers and implementing fine-grained access control, data forwarding is reduced, and bandwidth requirements are lowered through fog computing. Wang et al. [31] designed a road condition monitoring scheme based on cloud that incorporates a hierarchical structure with a root authority (RA) and sub-authorities. This method improves response speed by using multiple sub-authorities and reduces the pressure on the root authority. The cloud server can quickly verify the validity of ciphertexts and categorize traffic condition reports based on equivalence classes to achieve batch processing of tasks. In order to resist DoS attacks and improve communication efficiency, Wei et al. [32] introduced a multi-TA scheme designed for privacy protection under specific conditions, employing fog computing to enhance communication efficiency and facilitate the revocation of identities of illegal vehicles. Yang et al. [33] proposed an anonymous certificateless aggregated signature encryption system for conditional privacy protection. This scheme aggregates the signed messages from neighboring vehicles into aggregate ciphertexts using fog nodes, and batch verifies them. This scheme avoids key escrow and pseudonym management. Fog-cloud-based VANETs can enhance system computing capacity and communication efficiency, and reduce pressure on TA. However, few schemes combine clouds and fog, forming a more scalable cloud-fog architecture.

In terms of combining with blockchain, Liu et al. [34] implemented conditional privacy protection using identity-based group signatures and managed vehicle reputation values using blockchain to identify the reliability of messages. In order to improve the efficiency of blockchain-based conditional privacy protection authentication schemes, Zhou et al. [35] proposed the use of knowledge signatures for identity verification to improve efficiency and eliminate the need for secure channels for key distribution. Yang et al. [36] proposed an access control scheme for partial data privacy in VANETs using function encryption. This scheme divides data access into offline and online stages to reduce online computation costs and improve efficiency. The blockchain is used to guarantee identity records and prevent data tampering. To meet the requirements of high mobility and real-time performance in VANETs, Lin et al. [37] used a one-time public key generation mechanism to generate anonymous public keys and used knowledge signatures for authentication. The anonymous public keys for data sharing can be generated and published on the blockchain in advance, improving the overall performance of the protocol. However, none of the above schemes consider the requirements of vehicle social networking, which motivated us to propose a dynamic privacy-preserving anonymous authentication scheme for condition-matching in fog-cloud-based VANETs.

## 3. Preliminary

### 3.1. Elliptic Curve Cryptosystem

The definition of an elliptic curve over the finite field $Z_{p}^{*}$ with prime order *p* is E:$y^{2}=x^{3}+ax+b\left(\;\mathrm{mod}\;p\right)$, where the condition $4a^{3}+27b^{2}\neq 0\;\mathrm{mod}\;p$ is satisfied. The group representation on the elliptic curve is defined as $G=\left\{\left(x,y\right)|y^{2}=x^{3}+ax+b,a,b\in Z_{p}^{*}\right\}\cup \left\{O\right\}$, where O is called the point at infinity [38].

### 3.2. Related Complexity Assumptions

The security of the proposed scheme relies on the following complexity assumptions.

- ***Computational Discrete Logarithm (DL) Assumption***: For a given generator *P* of group *G* and a point Q=aP∈G, there exists no polynomial-time algorithm capable of determining the integer $a\in Z_{p}^{*}$.
- ***Decisional Computational Diffie-Hellman (CDH) Assumption***: When provided with the tuple (P,aP,bP,cP) in group *G*, where $a,b,c\in Z_{p}^{*}$ are unknown, no polynomial-time algorithm can distinguish whether cP=abP or represents a random element in *G*.

## 4. Scheme Formulation

### 4.1. System Model

**Figure 1:**
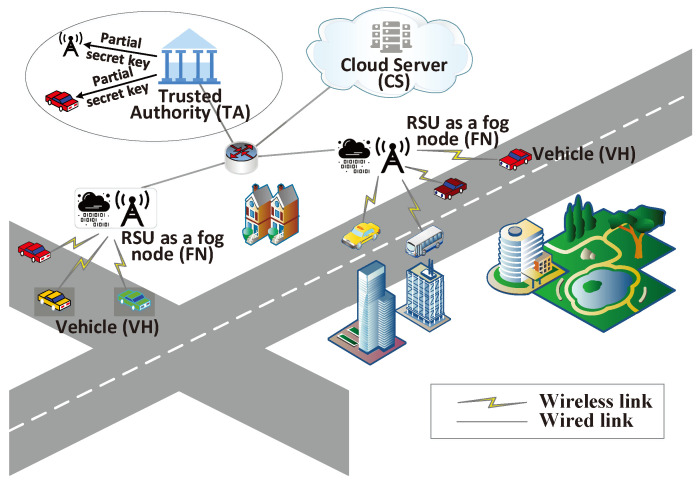
System architecture.

The system model is illustrated in Figure 1, and the key entities are introduced below.

- **Trusted Authority (TA)**: Responsible for global initialization and creating the main key pair of the system. Also, help to generate public and private key pairs for fog nodes to avoid key escrow issues. TA acts as the root TA and is not directly responsible for vehicle registration.
- **Fog Node (FN)**: Acts as a subordinate TA and registers with TA. Responsible for managing vehicle registration on the road segment. Adopts a certificateless approach to avoid vehicle key escrow issues.
- **Vehicle (VH)**: Participant in traffic. Vehicles register with fog nodes to obtain public-private key pairs. Vehicles can autonomously establish cross-domain groups based on traffic conditions and securely communicate within the group using negotiated secure keys.
- **Cloud Server (CS)**: Serves as the system’s data storage hub and message distribution center. Stores encrypted messages from vehicles and broadcasts them to other group members. Facilitates efficient communication and message retrieval.

Next, we will explain the system workflow in detail.

1. TA performs global initialization of the privacy-preserving vehicular communication system, creating the main key pair and other public parameters. TA securely stores the master key locally and publicly exposes the public parameters to other entities in the system.
2. The fog node (FN) registers with TA. FN chooses a random secret value and generates partial keys to send to TA along with its identity information. If FN is verified as legitimate, TA computes another partial key for FN and generates pseudonyms. FN combines its self-created partial key with the partial key generated by TA to form its final key pair.
3. The vehicle (VH) registers with FN. VH chooses a random secret value and generates partial keys to send to FH along with its identity information. If the information sent by VH is verified as legitimate, FN incorporates traffic conditions and a valid time period to generate partial keys and pseudonyms for VH. By combining its self-generated partial key with the partial key generated by FN, VH obtains a complete key pair.
4. When a group of vehicles (crossing fog nodes) wishes to establish a condition-based session group, they first use an anonymous authenticated key agreement to generate a group session key. Subsequently, the key obtained through negotiation is used to encrypt the sessions within the group.
5. During the communication phase, the message-sending vehicle transmits the message to the CS, which stores it and broadcasts it to other vehicles. Vehicles within the group can retrieve the complete encrypted message from the CS at any time.
6. When a vehicle applies to leave the existing group or a new vehicle joins the new group, the system recomputes and updates the group’s session key.

**Remark 1.**  *Crossing fog nodes refers to a vehicle registered at one RSU wishing to communicate with other vehicles registered at another RSU, which may be in a different city and crossing different fog nodes, in order to form a group.*

### 4.2. Security Requirements

The system needs to have the following functions and can provide a series of security protections.

- *Mutual authentication*: We select $VH_{\rho_{0},\theta_{0}}$ as the vehicle with higher computational power, while $VH_{\rho_{i},\theta_{i}}$ (1≤i≤n) represents vehicles with relatively weaker computational power. For the security of the group sessions based on traffic condition matching, mutual authentication between group members $VH_{\rho_{0},\theta_{0}}$ and $VH_{\rho_{i},\theta_{i}}$ becomes very important.
- *Fog node anonymity*: In order to eliminate some malicious users from obtaining the location information of vehicles through fog nodes, the scheme must generate pseudonyms for each fog node, and entities other than TA cannot obtain the real identity of fog nodes.
- *Vehicle anonymity*: In a socially attribute-enabled VANET, protecting the identity privacy of vehicles is crucial. A secure group session authentication key protocol should ensure the anonymity of vehicles, and entities other than TA cannot recover the real identity of vehicles from pseudonyms.
- *Fog node traceability*: When a malicious event involving a fog node (FN) is received, TA can obtain the real identity of FN from pseudonyms to achieve fog node traceability.
- *Vehicle traceability*: When malicious behavior of a vehicle (VH) is discovered, FN can use pseudonyms to obtain the real identity of VH to achieve vehicle traceability.
- *Session key establishment*: The communication key negotiation within the group is achieved through mutual authentication of all members in the group, creating a session key used to encrypt communication messages among vehicles in the group.
- *Cross-domain authenticated key agreement*: This scheme must allow the creation of groups between vehicles based on road conditions in different RSUs domains. Cross-domain vehicle groups are crucial for vehicle-based social topics.
- *Traffic condition matching*: Sharing VANETs-related traffic information is achieved by establishing groups based on traffic condition matching. Only vehicles encountering the same traffic conditions can negotiate a group session key. This traffic condition is invisible to potential attackers.
- *Time-limited keys*: The establishment of VANETs groups has temporary and spontaneous characteristics, so a time-limited key mechanism can ensure that vehicle keys automatically expire, improving security.
- *Perfect forward secrecy*: The scheme must have forward secrecy to ensure the confidentiality of intra-group communication in VANETs. Even if a malicious user gains knowledge of the group vehicles, they cannot derive the original group session key.
- *Resistance against replay attacks*: This scheme should be able to avoid the harm caused by replay attacks, where attackers repetitively send valid messages to vehicles, fog nodes, or TA.
- *Resistance against impersonation attacks*: The scheme should be able to resist impersonation attacks, where attackers pretend to be one of the entities involved in the scheme and send misleading information to the other communicating party.
- *Resistance against tampering attacks*: Prevent tampering attacks, where attackers secretly modify transmitted information in VANET communication without the knowledge of the communicating party.

### 4.3. Security Model

In this scheme, we will establish two categories of adversaries [39,40].

*Adversary I*: This category of adversary is represented as $A_{I}$. $A_{I}$ is unable to obtain the master key MSK of TA, but $A_{I}$ can query the public keys of fog nodes and vehicles, and $A_{I}$ has the ability to replace the public keys with forged ones. $A_{I}$ can freely query partial private keys and the secret values generated by FN and VH, or attempt to disrupt partial private keys and the secret values of FN and VH. The constraints for $A_{I}$ are: (1) $A_{I}$ cannot disrupt the challenger vehicles, (2) if the public keys of FN and VH are replaced, $A_{I}$ is not allowed to query the partial private keys of FN and VH or disrupt FN and VH. $A_{I}$ effectively simulates a malicious vehicle in the system.

*Adversary II*: This category of adversary is represented as $A_{\mathrm{II}}$, which has access to the master key MSK of TA. However, $A_{\mathrm{II}}$ does not replace the permissions of the VANETs vehicle public key. With the knowledge of TA’s master key MSK, $A_{\mathrm{II}}$ can compute the partial private keys of all vehicles. The constraint for $A_{\mathrm{II}}$ is not to disrupt the challenger vehicles. $A_{\mathrm{II}}$ can be conceptualized as a simulation of eavesdropping on TA.

Defined by an interactive game consisting of an adversary A and a challenger C, the security model of this scheme is established.

*Initialization*: In this phase, the challenger C first creates the system’s public parameters and master public key, then exposes it to the adversary A. If the adversary belongs to type $A_{I}$, C keeps the master key secret. If A is of type $A_{\mathrm{II}}$, C reveals the master key to A but restricts $A_{\mathrm{II}}$ adversaries from making substitution key requests in subsequent games.

*Query Phase*: During this phase, the adversary A can initiate various queries beyond constraints.

- *Hash Query*: A hash function $H_{i}$ and a message $m_{i}$ are specified by the adversary A to query the challenger C. The corresponding hash value is generated by the challenger C and returned to A.
- *Symmetric Encryption Query*: The adversary A initiates a symmetric encryption query using a symmetric key $k_{i}$ and a message $m_{i}$. The challenger C responds by providing the ciphertext $c_{i}$.
- *Extract Secret Value of* $FN_{\rho_{i}}$: The adversary A initiates a query for the secret value of fog node $FN_{\rho_{i}}$. In response, the challenger C discloses the secret value of $FN_{\rho_{i}}$ to A.
- *Extract Partial Key of* $FN_{\rho_{i}}$: The adversary A initiates queries to extract the partial secret key associated with fog node $FN_{\rho_{i}}$. In response, the challenger C discloses the partial secret key of $FN_{\rho_{i}}$ to A.
- *Request public key of* $FN_{\rho_{i}}$: Public keys are made accessible to adversaries. The adversary A initiates queries to extract the public key associated with fog node $FN_{\rho_{i}}$. In response, the challenger C provides the public key $PK_{FN_{\rho_{i}}}$ to A.
- *Replace public key of* $FN_{\rho_{i}}$: The adversary A has the capability to substitute $PK_{FN_{\rho_{i}}}$ with a carefully chosen valid public key replacement, denoted as $PK_{FN_{\rho_{i}}}^{\prime }$. It is important to note that the public key of the challenged fog node cannot undergo replacement, imposing a specific restriction.
- *Extract secret value from* $VH_{\rho_{i},\theta_{i}}$: The adversary A initiates queries to obtain the secret value associated with vehicle $VH_{\rho_{i},\theta_{i}}$. In response, the challenger C discloses the secret value of $VH_{\rho_{i},\theta_{i}}$ to A.
- *Extract partial secret key from* $VH_{\rho_{i},\theta_{i}}$: The adversary A initiates queries to obtain the partial secret key associated with vehicle $VH_{\rho_{i},\theta_{i}}$. In response, the challenger C discloses the partial secret key of $VH_{\rho_{i},\theta_{i}}$ to A.
- *Request public key of* $VH_{\rho_{i},\theta_{i}}$: Public keys are made accessible to adversaries. The adversary A initiates queries to obtain the public key associated with vehicle $VH_{\rho_{i},\theta_{i}}$. In response, the challenger C provides the public key $PK_{VH_{\rho_{i},\theta_{i}}}$ to A.
- *Replace public key of* $VH_{\rho_{i},\theta_{i}}$: The adversary A possesses the capability to substitute $PK_{VH_{\rho_{i},\theta_{i}}}$ with a carefully chosen valid public key replacement, denoted as $PK_{VH_{\rho_{i},\theta_{i}}}^{\prime }$. It is crucial to highlight that the public key of the challenged vehicle cannot undergo replacement, subject to specific restrictions.
- *Execute*: Upon receiving an execution request from A, the challenger C generates and returns the response information to A.
- *Reveal group authenticated key*: Upon receiving a query for the group authenticated key, the challenger C discloses the group authenticated key GSK to A.
- *Corrupt* $FN_{\rho_{i}}$: In response to the corruption query targeting fog node $FN_{\rho_{i}}$, the challenger C divulges the secret key $SK_{FN_{\rho_{i}}}$.
- *Corrupt* $VH_{\rho_{i},\theta_{i}}$: In response to the corruption query targeting vehicle $VH_{\rho_{i},\theta_{i}}$, the challenger C discloses the secret key $SK_{VH_{\rho_{i},\theta_{i}}}$.
- *Test*: In the Test phase, a coin *b* is randomly tossed by the challenger C from the set {0,1}. If *b* equals 1, C furnishes A with the genuine authentication information among the challenged vehicles. If *b* equals 0, randomly selected authentication information will be provided.

*Response*: Finally, the adversary A submits a guessed result $b^{\prime }$ to the challenger C. Should $b^{\prime }$ be equal to *b*, the adversary wins the game, and the advantage is computed as $Adv\left(A\right)=|Pr\left[b^{\prime }=b\right]-1/2|$.

**Definition 1.**  *This scheme’s security is contingent on the polynomial-time adversary A (of either type $A_{I}$ or $A_{\mathrm{II}}$) being unable to win the interactive game with a non-negligible advantage. In simpler terms, any polynomial-time adversary A that attains a non-negligible advantage Adv(A) in the game is deemed negligible.*

## 5. The Proposed System

In Table 1, we establish the primary symbols and terms utilized throughout this document. Following this, we detail the initial configuration of the system, the registration processes for both fog nodes and vehicles, the protocols for group key agreement, and the procedures for dynamic vehicle management. The verification of the system’s operational accuracy is presented in Supplemental Material A.

### 5.1. Initial Configuration Stage

The TA initiates the setup algorithm by taking the security parameter $\kappa \in Z^{+}$ as an input. This process results in the derivation of system parameters along with a key pair, consisting of the system’s master public and secret keys.

(1) Opting for an elliptic curve *E* over a finite field *p*, the TA makes a selection, where *G* represents the elliptic curve group and *P* is its generator.

(2) TA randomly chooses $x\in_{R}Z_{p}^{*}$ and calculates $P_{pub}=xP$. The system master secret key is MSK=x and master public key is $MPK=(P,P_{pub})$.

(3) For secure encryption/decryption, TA chooses a symmetric pair (SEnc/SDec) with a key space K. Additionally, TA chooses cryptographic hash functions $H_{0}:{\left\{0,1\right\}}^{*}\to K$ and $H_{i}:{\left\{0,1\right\}}^{*}\to Z_{p}^{*}$ (1≤i≤6) that are resistant to collusion.

(4) Publication by TA includes the master public key MPK and the system’s public parameters $(G,SEnc,SDec,H_{0},H_{1},$$\cdots ,H_{6})$. The master secret key MSK is retained in confidence by TA.

### 5.2. Fog Node Registration

In the pursuit of joining the system as the *i*-th fog node, $FN_{\rho_{i}}$ initiates its registration with TA. Upon receiving the registration request, TA undertakes a validation process to ascertain the functionality of $FN_{\rho_{i}}$ as an RSU. If the evaluation proves negative, the request is dismissed; however, in the affirmative case, TA and $FN_{\rho_{i}}$ engage in mutual collaboration to establish the key pair for $FN_{\rho_{i}}$. It is noteworthy that this key generation process operates in a key escrow-free and certificateless manner.

(1) **Set Secret Value**: The fog node $FN_{\rho_{i}}$ with identity $ID_{FN_{\rho_{i}}}$ selects $x_{FN_{\rho_{i}}}\in_{R}Z_{p}^{*}$ and computers $P_{FN_{\rho_{i}}}=x_{FN_{\rho_{i}}}P$. Upon determining the secret value, $FN_{\rho_{i}}$ designates $x_{FN_{\rho_{i}}}$ and conveys the pair $\left(ID_{FN_{\rho_{i}}},P_{FN_{\rho_{i}}}\right)$ to TA through a secure channel.

(2) **Partial Secret Key Extraction**: This algorithm takes TA’s master secret key MSK, $FN_{\rho_{i}}$’s identity $ID_{FN_{\rho_{i}}}$ and the public value $P_{FN_{\rho_{i}}}$ as input, it outputs $FN_{\rho_{i}}$’s partial secret key and pseudo identity.

- TA selects $\mu_{FN_{\rho_{i}}}\in_{R}Z_{p}^{*}$ and computes $FN_{\rho_{i}}$’s pseudo identity: $PID_{FN_{\rho_{i}}}=SEnc_{H_{0}\left(x\right)}\left(ID_{FN_{\rho_{i}}},\mu_{FN_{\rho_{i}}}\right).$
- TA chooses $r_{FN_{\rho_{i}}}\in_{R}Z_{p}^{*}$ and computes $R_{FN_{\rho_{i}}}=r_{FN_{\rho_{i}}}P,\;\alpha_{FN_{\rho_{i}}}=H_{1}\left(PID_{FN_{\rho_{i}}},P_{FN_{\rho_{i}}},R_{FN_{\rho_{i}}}\right).$
- TA calculates $y_{FN_{\rho_{i}}}=\alpha_{FN_{\rho_{i}}}x+r_{FN_{\rho_{i}}}$ and sends the partial secret key $y_{FN_{\rho_{i}}}$ to $FN_{\rho_{i}}$ via secure channel.
- Upon receiving $y_{FN_{\rho_{i}}}$, $FN_{\rho_{i}}$ verifies the equation
  (1) $$y_{FN_{\rho_{i}}}P=\alpha_{FN_{\rho_{i}}}P_{pub}+R_{FN_{\rho_{i}}}.$$
  The validity of the partial secret key $y_{FN_{\rho_{i}}}$ is contingent on the equation holding, and vice versa.

(3) **Set Secret Value**: The fog node $FN_{\rho_{i}}$, identified by the pseudo identity $PID_{FN_{\rho_{i}}}$, assigns $SK_{FN_{\rho_{i}}}=\left(x_{FN_{\rho_{i}}},y_{FN_{\rho_{i}}}\right)$ as its confidential secret key.

(4) **Set Public Key**: The fog node $FN_{\rho_{i}}$, associated with the pseudo identity $PID_{FN_{\rho_{i}}}$, designates $PK_{FN_{\rho_{i}}}=\left(P_{FN_{\rho_{i}}},R_{FN_{\rho_{i}}}\right)$ as its public key, accessible within the system.

### 5.3. Vehicle Reporting and Registration

A vehicle $VH_{\rho_{i},\theta_{i}}$ informs a fog node $FN_{\rho_{i}}$ about a traffic condition $TC_{VH_{\rho_{i},\theta_{i}}}\in \mathrm{TC}$. Subsequently, $FN_{\rho_{i}}$ and $VH_{\rho_{i},\theta_{i}}$ engage in an interaction to generate the public/secret key for $VH_{\rho_{i},\theta_{i}}$. Notably, this key generation procedure is designed to circumvent the key escrow problem. TA establishes a predefined expiration time $VT_{VH_{\rho_{i},\theta_{i}}}$ for the key pair of each vehicle. For example, if the key’s expiration time is set to 1 December 2023, at 14:30, it is represented as “202312011430”. Other vehicles can verify whether the key of that vehicle is within its validity period based on $VT_{VH_{\rho_{i},\theta_{i}}}$.

(1) **Set Secret Value**: The vehicle $VH_{\rho_{i},\theta_{i}}$ with identity $ID_{VH_{\rho_{i},\theta_{i}}}$ selects $x_{VH_{\rho_{i},\theta_{i}}}\in_{R}Z_{p}^{*}$ and computes $P_{VH_{\rho_{i},\theta_{i}}}=x_{VH_{\rho_{i},\theta_{i}}}P$. Then, $VH_{\rho_{i},\theta_{i}}$ sets $x_{VH_{\rho_{i},\theta_{i}}}$ as the secret value and securely transmits $\left(ID_{VH_{\rho_{i},\theta_{i}}},P_{VH_{\rho_{i},\theta_{i}}}\right)$ to $FN_{\rho_{i}}$ through the secure channel.

(2) **Partial Secret Key Extraction**: As input, $FN_{\rho_{i}}$’s secret key $SK_{FN_{\rho_{i}}}$, $VH_{\rho_{i},\theta_{i}}$’s identity $ID_{VH_{\rho_{i},\theta_{i}}}$, and the public value $P_{VH_{\rho_{i},\theta_{i}}}$ are taken by this algorithm. In turn, $VH_{\rho_{i},\theta_{i}}$’s pseudo-identity and partial secret key are outputted.

- $FN_{\rho_{i}}$ selects $\mu_{VH_{\rho_{i},\theta_{i}}}\in_{R}Z_{p}^{*}$ and computes $P_{VH_{\rho_{i},\theta_{i}}}$’s pseudo-identity: $PID_{VH_{\rho_{i},\theta_{i}}}=SEnc_{H_{0}\left(x_{FN_{\rho_{i}}},y_{FN_{\rho_{i}}}\right)}\left(ID_{VH_{\rho_{i},\theta_{i}}},\mu_{VH_{\rho_{i},\theta_{i}}}\right).$
- $FN_{\rho_{i}}$ chooses $r_{VH_{\rho_{i},\theta_{i}}}\in_{R}Z_{p}^{*}$ and computes $R_{VH_{\rho_{i},\theta_{i}}}=r_{VH_{\rho_{i},\theta_{i}}}P,\beta_{VH_{\rho_{i},\theta_{i}}}=H_{2}\left(PID_{FN_{\rho_{i}}},PID_{VH_{\rho_{i},\theta_{i}}},P_{VH_{\rho_{i},\theta_{i}}},R_{VH_{\rho_{i},\theta_{i}}},VT_{VH_{\rho_{i},\theta_{i}}},TC_{VH_{\rho_{i},\theta_{i}}}\right).$
- $FN_{\rho_{i}}$ calculates $y_{VH_{\rho_{i},\theta_{i}}}=\beta_{VH_{\rho_{i},\theta_{i}}}\left(x_{FN_{\rho_{i}}}+y_{FN_{\rho_{i}}}\right)+r_{VH_{\rho_{i},\theta_{i}}}$ and sends the partial secret key $y_{VH_{\rho_{i},\theta_{i}}}$ to $VH_{\rho_{i},\theta_{i}}$ via secure channel.
- Receiving $y_{VH_{\rho_{i},\theta_{i}}}$, the vehicle $VH_{\rho_{i},\theta_{i}}$ verifies whether the following equation is equal:
  (2) $$y_{VH_{\rho_{i},\theta_{i}}}P=\beta_{VH_{\rho_{i},\theta_{i}}}\left(P_{FN_{\rho_{i}}}+\alpha_{FN_{\rho_{i}}}P_{pub}+R_{FN_{\rho_{i}}}\right)+R_{VH_{\rho_{i},\theta_{i}}}.$$
  The validity of the partial secret key $y_{VH_{\rho_{i},\theta_{i}}}$ is contingent on the equation holding, and vice versa.

(3) **Set Secret Key**: The secret key $SK_{VH_{\rho_{i},\theta_{i}}}=\left(x_{VH_{\rho_{i},\theta_{i}}},y_{VH_{\rho_{i},\theta_{i}}}\right)$ is adopted by the vehicle $VH_{\rho_{i},\theta_{i}}$ and is confidentially stored.

(4) **Set Public Key**: Adopting $PK_{VH_{\rho_{i},\theta_{i}}}=\left(P_{VH_{\rho_{i},\theta_{i}}},R_{VH_{\rho_{i},\theta_{i}}},VT_{VH_{\rho_{i},\theta_{i}}}\right)$ as its public key, the vehicle $VH_{\rho_{i},\theta_{i}}$ makes this information public within the system.

### 5.4. Condition-Matching-Based Authenticated Key Agreement

Assuming the vehicles $V_{0}=\left\{VH_{\rho_{1},\theta_{1}},\cdots \right\}$ and $VH_{\rho_{0},\theta_{0}}$ aim to establish a secure group communication based on condition-matching, ensuring the security of their traffic discussions. The first step involves establishing a group session key. In this scenario, vehicle $VH_{\rho_{0},\theta_{0}}$ possesses relatively robust computational capabilities, while the vehicles within $V_{0}$ have lower computational power. The group-authenticated key agreement unfolds through the following interactive steps.

**Mutual Authentication Requests Within the Group**:

The powerful vehicle $VH_{\rho_{0},\theta_{0}}$ sends $\left(PID_{FN_{\rho_{0}}},PID_{VH_{\rho_{0},\theta_{0}}}\right)$ to $V_{0}$, and $VH_{\rho_{i},\theta_{i}}\in V_{0}$ sends $\left(PID_{FN_{\rho_{i}}},PID_{VH_{\rho_{i},\theta_{i}}}\right)$ to $VH_{\rho_{0},\theta_{0}}$.

Receiving the messages $\left(PID_{FN_{\rho_{0}}},PID_{VH_{\rho_{0},\theta_{0}}}\right)$, the vehicle $VH_{\rho_{i},\theta_{i}}\in V_{0}$ chooses $a_{VH_{\rho_{i},\theta_{i}}}\in_{R}Z_{p}^{*}$ and computes $A_{VH_{\rho_{i},\theta_{i}}}=a_{VH_{\rho_{i},\theta_{i}}}\cdot P,b_{VH_{\rho_{i},\theta_{i}}}=\left(a_{VH_{\rho_{i},\theta_{i}}}+\gamma_{VH_{\rho_{i},\theta_{i}}}+x_{VH_{\rho_{i},\theta_{i}}}+y_{VH_{\rho_{i},\theta_{i}}}\right)\cdot P,\Gamma_{VH_{\rho_{i},\theta_{i}}}=\;a_{VH_{\rho_{i},\theta_{i}}}y_{VH_{\rho_{i},\theta_{i}}}\cdot P+\left[P_{VH_{\rho_{0},\theta_{0}}}+\beta_{VH_{\rho_{0},\theta_{0}}}\left(P_{FN_{\rho_{0}}}+\alpha_{FN_{\rho_{0}}}P_{pub}+R_{FN_{\rho_{0}}}\right)+R_{VH_{\rho_{0},\theta_{0}}}\right],$ where $\alpha_{FN_{\rho_{0}}}=H_{1}\left(PID_{FN_{\rho_{0}}},P_{FN_{\rho_{0}}},R_{FN_{\rho_{0}}}\right),\beta_{VH_{\rho_{0},\theta_{0}}}=H_{2}\left(PID_{FN_{\rho_{0}}},PID_{VH_{\rho_{0},\theta_{0}}},P_{VH_{\rho_{0},\theta_{0}}},R_{VH_{\rho_{0},\theta_{0}}},\;VT_{VH_{\rho_{0},\theta_{0}}},TC_{VH_{\rho_{i},\theta_{i}}}\right),\gamma_{VH_{\rho_{i},\theta_{i}}}=H_{3}\left(A_{VH_{\rho_{i},\theta_{i}}},P_{VH_{\rho_{i},\theta_{i}}},R_{VH_{\rho_{i},\theta_{i}}},VT_{VH_{\rho_{i},\theta_{i}}},TC_{VH_{\rho_{i},\theta_{i}}}\right).$

Then, $VH_{\rho_{i},\theta_{i}}$ sends $\left(A_{VH_{\rho_{i},\theta_{i}}},b_{VH_{\rho_{i},\theta_{i}}},\Gamma_{VH_{\rho_{i},\theta_{i}}}\right)$ to $VH_{\rho_{0},\theta_{0}}$, for 1≤i≤n.

**Authentication Process for High-Computational-Power Vehicles**:

When the vehicle $VH_{\rho_{0},\theta_{0}}$ receives messages $\left(A_{VH_{\rho_{i},\theta_{i}}},b_{VH_{\rho_{i},\theta_{i}}},\Gamma_{VH_{\rho_{i},\theta_{i}}}\right)$ from each vehicle $VH_{\rho_{i},\theta_{i}}\in V_{0}$, $VH_{\rho_{0},\theta_{0}}$ verifies whether $b_{VH_{\rho_{i},\theta_{i}}}-\left(A_{VH_{\rho_{i},\theta_{i}}}+\gamma_{VH_{\rho_{i},\theta_{i}}}P\right)=P_{VH_{\rho_{i},\theta_{i}}}+\beta_{VH_{\rho_{i},\theta_{i}}}\left(P_{FN_{\rho_{i}}}+\alpha_{FN_{\rho_{i}}}P_{pub}+R_{FN_{\rho_{i}}}\right)+R_{VH_{\rho_{i},\theta_{i}}}$, where $\alpha_{FN_{\rho_{i}}}=H_{1}\left(PID_{FN_{\rho_{i}}},P_{FN_{\rho_{i}}},R_{FN_{\rho_{i}}}\right),\beta_{VH_{\rho_{i},\theta_{i}}}=H_{2}\left(PID_{FN_{\rho_{i}}},PID_{VH_{\rho_{i},\theta_{i}}},P_{VH_{\rho_{i},\theta_{i}}},R_{VH_{\rho_{i},\theta_{i}}},\;VT_{VH_{\rho_{i},\theta_{i}}},TC_{VH_{\rho_{0},\theta_{0}}}\right),\gamma_{VH_{\rho_{i},\theta_{i}}}=H_{3}$$(A_{VH_{\rho_{i},\theta_{i}}},P_{VH_{\rho_{i},\theta_{i}}},R_{VH_{\rho_{i},\theta_{i}}},VT_{VH_{\rho_{i},\theta_{i}}},TC_{VH_{\rho_{0},\theta_{0}}}).$

If the above equation holds true, it indicates that the identity of $VH_{\rho_{i},\theta_{i}}$ has been verified and $VH_{\rho_{i},\theta_{i}}$ encounters the same traffic condition as $VH_{\rho_{0},\theta_{0}}$. Suppose the verified vehicle set be $U=\{VH_{\rho_{1},\theta_{1}},\cdots ,VH_{\rho_{n},\theta_{n}}\}$. $VH_{\rho_{0},\theta_{0}}$ sets $PID_{U}=PID_{VH_{\rho_{1},\theta_{1}}}||\cdots ||PID_{VH_{\rho_{n},\theta_{n}}}$.

Then, $VH_{\rho_{0},\theta_{0}}$ chooses $a_{VH_{\rho_{0},\theta_{0}}}\in_{R}Z_{p}^{*}$ and computes $\Gamma_{VH_{\rho_{i},\theta_{i}}}^{\prime }=a_{VH_{\rho_{0},\theta_{0}}}y_{VH_{\rho_{0},\theta_{0}}}\cdot P+\left(x_{VH_{\rho_{0},\theta_{0}}}+y_{VH_{\rho_{0},\theta_{0}}}\right)\cdot P-\Gamma_{VH_{\rho_{i},\theta_{i}}},\Gamma_{U}=\sum_{VH_{\rho_{i},\theta_{i}}\in U}\Gamma_{VH_{\rho_{i},\theta_{i}}}^{\prime }\;K_{VH_{\rho_{0},\theta_{0}}}=H_{4}\left(\Gamma_{U}\right)\cdot P,GSK=H_{5}\left(PID_{U},PID_{0},TC_{VH_{\rho_{0},\theta_{0}}},K_{VH_{\rho_{0},\theta_{0}}}\right),Z_{VH_{\rho_{i},\theta_{i}}}=H_{4}\left(\Gamma_{U}\right)\cdot P\;+r_{VH_{\rho_{0},\theta_{0}}}A_{VH_{\rho_{i},\theta_{i}}},\Lambda_{VH_{\rho_{i},\theta_{i}}}=a_{VH_{\rho_{0},\theta_{0}}}y_{VH_{\rho_{0},\theta_{0}}}\cdot P+\left[P_{VH_{\rho_{i},\theta_{i}}}+\beta_{VH_{\rho_{i},\theta_{i}}}\left(P_{FN_{\rho_{i}}}+\alpha_{FN_{\rho_{i}}}P_{pub}+\;R_{FN_{\rho_{i}}}\right)+R_{VH_{\rho_{i},\theta_{i}}}\right],Auth_{0,i}=H_{6}\left(PID_{U},\Lambda_{VH_{\rho_{i},\theta_{i}}},\Gamma_{VH_{\rho_{i},\theta_{i}}},\Gamma_{VH_{\rho_{i},\theta_{i}}}^{\prime },K_{VH_{\rho_{0},\theta_{0}}}\right),$ where $\alpha_{FN_{\rho_{i}}}=H_{1}\left(PID_{FN_{\rho_{i}}},P_{FN_{\rho_{i}}},R_{FN_{\rho_{i}}}\right),\beta_{VH_{\rho_{i},\theta_{i}}}=H_{2}\left(PID_{FN_{\rho_{i}}},PID_{VH_{\rho_{i},\theta_{i}}},P_{VH_{\rho_{i},\theta_{i}}},R_{VH_{\rho_{i},\theta_{i}}},VT_{VH_{i,i}},\;TC_{VH_{\rho_{0},\theta_{0}}}\right).$

Then, $VH_{\rho_{0},\theta_{0}}$ sends $\left(Auth_{0,i},PID_{U},Z_{VH_{\rho_{i},\theta_{i}}},\Lambda_{VH_{\rho_{i},\theta_{i}}}\right)$ to $VH_{\rho_{i},\theta_{i}}\in U$.

**Authentication Process for Low-Computational-Power Vehicles**:

Receiving $\left(Auth_{0,i},PID_{U},Z_{VH_{\rho_{i},\theta_{i}}},\Lambda_{VH_{\rho_{i},\theta_{i}}}\right)$ from $VH_{\rho_{0},\theta_{0}}$, each vehicle $VH_{\rho_{i},\theta_{i}}\in U$ computes $Auth_{i,0}=H_{6}\left(PID_{U},\Lambda_{VH_{\rho_{i},\theta_{i}}},\Gamma_{VH_{\rho_{i},\theta_{i}}},\Lambda_{VH_{\rho_{i},\theta_{i}}}^{\prime },K_{VH_{\rho_{i},\theta_{i}}}\right),$ where $\Lambda_{VH_{\rho_{i},\theta_{i}}}^{\prime }=\Lambda_{VH_{\rho_{i},\theta_{i}}}-\left[a_{VH_{\rho_{i},\theta_{i}}}y_{VH_{\rho_{i},\theta_{i}}}\cdot P+\left(x_{VH_{\rho_{i},\theta_{i}}}+y_{VH_{\rho_{i},\theta_{i}}}\right)\cdot P\right],K_{VH_{\rho_{i},\theta_{i}}}=Z_{VH_{\rho_{i},\theta_{i}}}-\;\left(a_{VH_{\rho_{i},\theta_{i}}}\right)\cdot R_{VH_{\rho_{0},\theta_{0}}}.$

If $Auth_{i,0}=Auth_{0,i}$, it ensures that the identity of $VH_{\rho_{0},\theta_{0}}$ is authenticated and $VH_{\rho_{0},\theta_{0}}$ encounters the same traffic condition as $VH_{\rho_{i},\theta_{i}}$. Then, $VH_{\rho_{i},\theta_{i}}$ computes the group session key $GSK=H_{5}\left(PID_{U},PID_{0},TC_{VH_{\rho_{i},\theta_{i}}},K_{VH_{\rho_{i},\theta_{i}}}\right).$

### 5.5. Vehicle Join

If a set of vehicles $U_{0}^{\prime }=\left\{VH_{n+1},\cdots ,VH_{h}\right\}$ with lower computational power encounters the same traffic condition and desires to join the existing session group, the current group members collaboratively establish a new group authentication key as follows.

**Mutual Authentication Requests Within the Group**:

The vehicle $VH_{\rho_{0},\theta_{0}}$ with relatively robust computational capabilities sends $(PID_{FN_{\rho_{0}}},$$PID_{VH_{\rho_{0},\theta_{0}}})$ to $U_{0}^{\prime }$, and $VH_{\rho_{i},\theta_{i}}\in U^{\prime }$ sends $\left(PID_{FN_{\rho_{i}}},PID_{VH_{\rho_{i},\theta_{i}}}\right)$ to $VH_{\rho_{0},\theta_{0}}$.

Receiving the messages $\left(PID_{FN_{\rho_{0}}},PID_{VH_{\rho_{0},\theta_{0}}}\right)$, the vehicle $VH_{\rho_{i},\theta_{i}}\in U_{0}^{\prime }$ chooses $a_{VH_{\rho_{i},\theta_{i}}}\in_{R}Z_{p}^{*}$ and computes $\left(A_{VH_{\rho_{i},\theta_{i}}},b_{VH_{\rho_{i},\theta_{i}}},\Gamma_{VH_{\rho_{i},\theta_{i}}}\right)$ as in the Section 5.4, which is then sent to $VH_{\rho_{0},\theta_{0}}$, for n+1≤i≤h.

**Authentication Process for High-Computational-Power Vehicles**:

Upon receiving messages $\left(A_{VH_{\rho_{i},\theta_{i}}},b_{VH_{\rho_{i},\theta_{i}}},\Gamma_{VH_{\rho_{i},\theta_{i}}}\right)$ from each vehicle $VH_{\rho_{i},\theta_{i}}\in U_{0}^{\prime }$, the vehicle $VH_{\rho_{0},\theta_{0}}$ verifies $b_{VH_{\rho_{i},\theta_{i}}}$ as outlined in Section 5.4. It is assumed that these vehicles are all authenticated to be genuine and share the same traffic condition. $VH_{\rho_{0},\theta_{0}}$ sets $U^{\prime }=U\cup U_{0}^{\prime }=\left\{VH_{\rho_{1},\theta_{1}},\cdots ,VH_{\rho_{h},\theta_{h}}\right\}$ and $PID_{U^{\prime }}=PID_{U}\cup PID_{U_{0}^{\prime }}=PID_{VH_{\rho_{1},\theta_{1}}}||\cdots ||PID_{VH_{\rho_{h},\theta_{h}}}$.

Then, $VH_{\rho_{0},\theta_{0}}$ chooses $a_{VH_{\rho_{0},\theta_{0}}}^{\prime }\in_{R}Z_{p}^{*}$ and computes $\Gamma_{VH_{\rho_{i},\theta_{i}}}^{\prime \prime }=a_{VH_{\rho_{0},\theta_{0}}}^{\prime }y_{VH_{\rho_{0},\theta_{0}}}\cdot P+\left(x_{VH_{\rho_{0},\theta_{0}}}+y_{VH_{\rho_{0},\theta_{0}}}\right)\cdot P-\Gamma_{VH_{\rho_{i},\theta_{i}}},\Gamma_{U^{\prime }}=\sum_{VH_{\rho_{i},\theta_{i}}\in U^{\prime }}\Gamma_{VH_{\rho_{i},\theta_{i}}}^{\prime \prime }\;K_{VH_{\rho_{0},\theta_{0}}}^{\prime }=H_{4}\left(\Gamma_{U^{\prime }}\right)\cdot P,GSK^{\prime }=H_{5}\left(PID_{U^{\prime }},PID_{0},TC_{VH_{\rho_{0},\theta_{0}}},K_{VH_{\rho_{0},\theta_{0}}}^{\prime }\right),Z_{VH_{\rho_{i},\theta_{i}}}^{\prime }=H_{4}\left(\Gamma_{U^{\prime }}\right)\cdot P+\;r_{VH_{\rho_{0},\theta_{0}}}A_{VH_{\rho_{i},\theta_{i}}},\Lambda_{VH_{\rho_{i},\theta_{i}}}^{\prime }=a_{VH_{\rho_{0},\theta_{0}}}^{\prime }y_{VH_{\rho_{0},\theta_{0}}}\cdot P+\left[P_{VH_{\rho_{i},\theta_{i}}}+\beta_{VH_{\rho_{i},\theta_{i}}}\left(P_{FN_{\rho_{i}}}+\alpha_{FN_{\rho_{i}}}P_{pub}\;+R_{FN_{\rho_{i}}}\right)+R_{VH_{\rho_{i},\theta_{i}}}\right],Auth_{0,i}^{\prime }=H_{6}\left(PID_{U^{\prime }},\Lambda_{VH_{\rho_{i},\theta_{i}}},\Gamma_{VH_{\rho_{i},\theta_{i}}},\Gamma_{VH_{\rho_{i},\theta_{i}}}^{\prime \prime },K_{VH_{\rho_{0},\theta_{0}}}^{\prime }\right),$ where $\alpha_{FN_{\rho_{i}}}=H_{1}\left(PID_{FN_{\rho_{i}}},P_{FN_{\rho_{i}}},R_{FN_{\rho_{i}}}\right),\beta_{VH_{\rho_{i},\theta_{i}}}=H_{2}\left(PID_{FN_{\rho_{i}}},PID_{VH_{\rho_{i},\theta_{i}}},P_{VH_{\rho_{i},\theta_{i}}},R_{VH_{\rho_{i},\theta_{i}}},VT_{VH_{i,i}},\;TC_{VH_{\rho_{0},\theta_{0}}}\right).$

Then, $VH_{\rho_{0},\theta_{0}}$ sends $\left(Auth_{0,i}^{\prime },PID_{U^{\prime }},Z_{VH_{\rho_{i},\theta_{i}}}^{\prime },\Lambda_{VH_{\rho_{i},\theta_{i}}}^{\prime }\right)$ to $VH_{\rho_{i},\theta_{i}}\in U^{\prime }$.

**Authentication Process for Low-Computational-Power Vehicles**:

Receiving $\left(Auth_{0,i}^{\prime },PID_{U^{\prime }},Z_{VH_{\rho_{i},\theta_{i}}}^{\prime },\Lambda_{VH_{\rho_{i},\theta_{i}}}^{\prime }\right)$ from $VH_{\rho_{0},\theta_{0}}$, each vehicle $VH_{\rho_{i},\theta_{i}}\in U^{\prime }$ computes $Auth_{i,0}^{\prime }=H_{6}\left(PID_{U^{\prime }},\Lambda_{VH_{\rho_{i},\theta_{i}}},\Gamma_{VH_{\rho_{i},\theta_{i}}},\Lambda_{VH_{\rho_{i},\theta_{i}}}^{\prime \prime },K_{VH_{\rho_{i},\theta_{i}}}^{\prime }\right),$ where $\Lambda_{VH_{\rho_{i},\theta_{i}}}^{\prime \prime }=\Lambda_{VH_{\rho_{i},\theta_{i}}}^{\prime }-\left[a_{VH_{\rho_{i},\theta_{i}}}y_{VH_{\rho_{i},\theta_{i}}}\cdot P+\left(x_{VH_{\rho_{i},\theta_{i}}}+y_{VH_{\rho_{i},\theta_{i}}}\right)\cdot P\right],K_{VH_{\rho_{i},\theta_{i}}}^{\prime }=Z_{VH_{\rho_{i},\theta_{i}}}^{\prime }-\;\left(a_{VH_{\rho_{i},\theta_{i}}}\right)\cdot R_{VH_{\rho_{0},\theta_{0}}}.$

If $Auth_{0,i}^{\prime }=Auth_{i,0}^{\prime }$, it indicates that the identity of $VH_{\rho_{0},\theta_{0}}$ is authenticated and $VH_{\rho_{0},\theta_{0}}$ has encountered the same traffic condition as $VH_{\rho_{i},\theta_{i}}\in U^{\prime }$. Then, $VH_{\rho_{i},\theta_{i}}$ computes the group session key $GSK^{\prime }=H_{5}\left(PID_{U^{\prime }},PID_{0},TC_{VH_{\rho_{i},\theta_{i}}},K_{VH_{\rho_{i},\theta_{i}}}^{\prime }\right).$

### 5.6. Vehicle Leave

If a set of vehicles $U_{0}^{\prime \prime }=\left\{VH_{j+1},\cdots ,VH_{n}\right\}$ wishes to exit the session group, the remaining group members collaborate to create a new group authenticated key as follows.

$VH_{\rho_{0},\theta_{0}}$ sets $U^{\prime \prime }=U\setminus U_{0}^{\prime \prime }=\left\{VH_{\rho_{1},\theta_{1}},\cdots ,VH_{\rho_{j},\theta_{j}}\right\}$ and $PID_{U^{\prime \prime }}=PID_{U}\setminus PID_{U_{0}^{\prime \prime }}=PID_{VH_{\rho_{1},\theta_{1}}}||\cdots ||PID_{VH_{\rho_{j},\theta_{j}}}$. Then, $VH_{\rho_{0},\theta_{0}}$ chooses $a_{VH_{\rho_{0},\theta_{0}}}^{\prime \prime }\in_{R}Z_{p}^{*}$ and computes $\Gamma_{VH_{\rho_{i},\theta_{i}}}^{\prime \prime \prime }=a_{VH_{\rho_{0},\theta_{0}}}^{\prime \prime }y_{VH_{\rho_{0},\theta_{0}}}\cdot P+\left(x_{VH_{\rho_{0},\theta_{0}}}+y_{VH_{\rho_{0},\theta_{0}}}\right)\cdot P-\Gamma_{VH_{\rho_{i},\theta_{i}}},\Gamma_{U^{\prime \prime }}=\sum_{VH_{\rho_{i},\theta_{i}}\in U}\Gamma_{VH_{\rho_{i},\theta_{i}}}^{\prime \prime \prime }\;K_{VH_{\rho_{0},\theta_{0}}}^{\prime \prime }=H_{4}\left(\Gamma_{U^{\prime \prime }}\right)\cdot P,GSK^{\prime \prime }=H_{5}\left(PID_{U^{\prime \prime }},PID_{0},TC_{VH_{\rho_{0},\theta_{0}}},K_{VH_{\rho_{0},\theta_{0}}}^{\prime \prime }\right),Z_{VH_{\rho_{i},\theta_{i}}}^{\prime \prime }=H_{4}\left(\Gamma_{U^{\prime \prime }}\right)\cdot P\;+r_{VH_{\rho_{0},\theta_{0}}}\cdot A_{VH_{\rho_{i},\theta_{i}}}^{\prime \prime \prime },\Lambda_{VH_{\rho_{i},\theta_{i}}}^{\prime \prime }=a_{VH_{\rho_{0},\theta_{0}}}^{\prime \prime }y_{VH_{\rho_{0},\theta_{0}}}\cdot P+\left[P_{VH_{\rho_{i},\theta_{i}}}+\beta_{VH_{\rho_{i},\theta_{i}}}\left(P_{FN_{\rho_{i}}}+\alpha_{FN_{\rho_{i}}}P_{pub}\;+R_{FN_{\rho_{i}}}\right)+R_{VH_{\rho_{i},\theta_{i}}}\right],Auth_{0,i}^{\prime \prime }=H_{6}\left(PID_{U^{\prime \prime }},\Lambda_{VH_{\rho_{i},\theta_{i}}},\Gamma_{VH_{\rho_{i},\theta_{i}}},\Gamma_{VH_{\rho_{i},\theta_{i}}}^{\prime \prime \prime },K_{VH_{\rho_{0},\theta_{0}}}^{\prime \prime }\right).$

Then, $VH_{\rho_{0},\theta_{0}}$ sends $\left(Auth_{0,i}^{\prime \prime },PID_{U^{\prime \prime }},Z_{VH_{\rho_{i},\theta_{i}}}^{\prime \prime },\Lambda_{VH_{\rho_{i},\theta_{i}}}^{\prime \prime }\right)$ to $VH_{\rho_{i},\theta_{i}}\in U^{\prime \prime }$.

Receiving $\left(Auth_{0,i}^{\prime \prime },PID_{U^{\prime \prime }},Z_{VH_{\rho_{i},\theta_{i}}}^{\prime \prime },\Lambda_{VH_{\rho_{i},\theta_{i}}}^{\prime \prime }\right)$ from $VH_{\rho_{0},\theta_{0}}$, each vehicle $VH_{\rho_{i},\theta_{i}}\in U^{\prime \prime }$ computes $Auth_{i,0}^{\prime }=H_{6}\left(PID_{U^{\prime \prime }},\Lambda_{VH_{\rho_{i},\theta_{i}}},\Gamma_{VH_{\rho_{i},\theta_{i}}},\Lambda_{VH_{\rho_{i},\theta_{i}}}^{\prime \prime \prime },K_{VH_{\rho_{i},\theta_{i}}}^{\prime \prime }\right),$ where $\Lambda_{VH_{\rho_{i},\theta_{i}}}^{\prime \prime \prime }=\Lambda_{VH_{\rho_{i},\theta_{i}}}^{\prime \prime }-\left[a_{VH_{\rho_{i},\theta_{i}}}y_{VH_{\rho_{i},\theta_{i}}}\cdot P+\left(x_{VH_{\rho_{i},\theta_{i}}}+y_{VH_{\rho_{i},\theta_{i}}}\right)\cdot P\right],K_{VH_{\rho_{i},\theta_{i}}}^{\prime \prime }=Z_{VH_{\rho_{i},\theta_{i}}}^{\prime \prime }-\;\left(a_{VH_{\rho_{i},\theta_{i}}}\right)\cdot R_{VH_{\rho_{0},\theta_{0}}}.$

If $Auth_{i,0}^{\prime \prime }=Auth_{0,i}^{\prime \prime }$, the vehicle $VH_{\rho_{i},\theta_{i}}\in U^{\prime \prime }$ obtains the negotiated group session key as $GSK^{\prime \prime }=H_{5}\left(PID_{U^{\prime \prime }},PID_{0},TC_{VH_{\rho_{i},\theta_{i}}},K_{VH_{\rho_{i},\theta_{i}}}^{\prime \prime }\right).$

## 6. Security Proof

**Theorem 1.**  *Assuming the decisional CDH assumption holds in the random oracle model, then the scheme we propose is secure against $A_{I}$ adversary (as defined in Section 4.3).*

The formal security proof of Theorem 1 is deferred to Supplemental Material B.

**Theorem 2.**  *In the scenario where the decisional CDH assumption is satisfied, the proposed group authenticated key agreement ensures security against $A_{\mathrm{II}}$ adversaries in the random oracle model.*

**Proof.**  The proof of Theorem 1 is followed with the following modification: the master key MSK=x can be obtained by a $A_{\mathrm{II}}$ attacker, but the attacker is not permitted to issue substitute public key queries. The remaining part of the proof remains unchanged. □

**Theorem 3.**  *The proposed system satisfies mutual authentication, fog node anonymity, vehicle anonymity, vehicle traceability, cross-domain authenticated key management, group key establishment, condition-matching, time-limited keys, perfect forward secrecy, impersonation/modification/ replay attack resistance.*

The proposed system meets the functional and security requirements defined in Section 4.2, which are proven in Supplemental Material C.

## 7. Performance Comparison and Analysis

To assess the performance of other existing conditional privacy-preserving schemes, a comparison will be made with the proposed system. Subsequently, an assessment of the computational and communication overheads of these schemes will be conducted in a real experimental environment.

### 7.1. Theoretical Analysis

Before conducting the comparison, we have defined certain symbols in Table 2. Our proposed system will be compared to the schemes introduced in [6,17,21]. We assume the vehicle group has a size of *n*.

#### 7.1.1. Analysis of Computation Overhead

We conducted a theoretical analysis of the computational expenses associated with these schemes in Table 3. When analyzing the computational costs, we do not include the overhead of global initialization and server registration (such as cloud and fog servers) as they are constant and do not vary with the number of vehicles. Additionally, the computational cost of ECC scalar addition is very low, so it is also not taken into account. In the computational analysis, for ease of understanding, we consider the total computational cost of a vehicle from registration to completing verification.

- Ma et al. [6] adopts ECC algorithm design, where the initiating vehicle performs four scalar multiplications on *G* and five computations hashed to $Z_{p}$. Hence, the computational overhead for the initiating vehicle is $3T_{M}+4T_{HZ}$. The verification task is accomplished through collaboration among the cloud server and fog nodes. Fog nodes execute four scalar multiplications on *G*, two computations hashed to $Z_{p}$, while the cloud server executes eight scalar multiplications on *G* and nine computations hashed to $Z_{p}$. Consequently, the total verification task requires $12T_{M}+13T_{HZ}$.
- In Xiong et al. [17], before a group session, the TA initially constructs an instance of the Chinese Remainder Theorem and finds a root. Each joining vehicle needs to perform 2l+3 scalar multiplications on *G* and l+4 computations hashed to $Z_{p}$. Thus, the total computational overhead for the authentication initiation phase is $1T_{cr}+\left(2l+3\right)T_{M}+\left(l+4\right)T_{HZ}$. The verification task requires five scalar multiplications on *G*, totaling $5T_{M}$.
- In Luo et al. [21], the initiating vehicle performs five scalar multiplications on G1, four computations hashed to $Z_{p}$, and 2n exponentiations on GT. Hence, the computational overhead for the initiating vehicle is $5T_{M}+4T_{HZ}+2nT_{PE}$. The verification task requires executing n+1 scalar multiplications on G1, two computations hashed to $Z_{p}$, *n* exponentiations on GT, and two bilinear pairing computations, resulting in a total verification task cost of $\left(n+1\right)T_{M}+2T_{HZ}+nT_{PE}+2T_{P}$.
- Our scheme eliminates time-consuming bilinear pairing computations. The computational overhead for the initiating vehicle is $12T_{M}+6T_{HZ}$, which does not exhibit linear growth with an increase in-group members. For verification, vehicles perform seven scalar multiplications on *G* and nine computations hashed to $Z_{p}$, totaling $7T_{M}+9T_{HZ}$ to establish a group authenticated session key.

**Table 3:** Comparison of computation overhead [6,17,21].

Scheme	Registration Phase	Authentication Phase
Ma et al.	$1T_{M}+1T_{HZ}$	$15T_{M}+17T_{HZ}$
Xiong et al.	$\left(2l+2\right)T_{M}+\left(l+1\right)T_{HZ}$	$1T_{cc}+1T_{cr}+7T_{M}+3T_{HZ}$
Luo et al.	$1T_{P}+1T_{M}+1T_{HG}$	$2T_{P}+3nT_{PP}+\left(n+6\right)T_{M}+6T_{HZ}$
Ours	$4T_{M}+2T_{HZ}$	$15T_{M}+13T_{HZ}$

*l*: the quantity of pseudo-identities generated for a vehicle in Xiong et al. [17]. *n*: the number of vehicles within the group.

#### 7.1.2. Analysis of Communication Overhead

In schemes [6,17], and our scheme, the session key is generated through negotiation, making subsequent communication processes dependent only on the length of the message itself. However, in Luo et al. [21], due to the employment of ring signatures, each communication involves additional data transmission. The communication process involves both sending and receiving parties; therefore, we uniformly consider the data quantity sent by the sender for computation. The theoretical analysis of communication overhead can be found in Table 4.

- In Ma et al. [6], utilizing offline key distribution, the communication cost for the initiating authenticated vehicle comprises three *G* elements and two $Z_{p}$ elements. The verification process necessitates the transfer of 13 *G* elements and seven $Z_{p}$ elements, resulting in a total communication cost of $16|G|+9|Z_{p}|$ for the authentication process.
- In Xiong et al. [17], the communication cost for the initiating authenticated vehicle during the registration phase is $2|Z_{p}\left|+l(2|G|+3\right|Z_{p}\left|\right)$, and during the authentication phase is $1|G|+4|Z_{p}\left|+|M\right|$. The overall communication cost sums up to $1|G|+6|Z_{p}\left|+l(2|G|+3\right|Z_{p}\left|\right)+\left|M\right|$.
- In Luo et al. [21], the communication cost for the initiating authenticated vehicle during the registration phase is $2|Z_{p}|$, and during the authentication phase is $\left(n+1\right)|G|+\left(n+2\right)|Z_{p}\left|+|M\right|$. The total communication cost is $\left(n+1\right)|G|+\left(n+4\right)|Z_{p}\left|+|M\right|$.
- In our scheme, the communication cost during the registration phase for the initiating authenticated vehicle is $1|G|+2|Z_{p}|$, during the initiation of authentication is $2|G|+4|Z_{p}|$, during verification is 3|G|, resulting in a total communication cost of $6|G|+6|Z_{p}|$.

In Luo et al. [21], with each communication involving the group size *n*, the communication cost becomes maximal. In [6,17], and our proposal, the registration and authentication times for each vehicle are not affected by other members.

**Table 4:** Comparison of communication overhead [6,17,21].

Scheme	Registration Phase	Authentication Phase
Ma et al.	$1|Z_{p}|$	$16|G|+9|Z_{p}|$
Xiong et al.	$2|Z_{p}\left|+l(2|G|+3\right|Z_{p}\left|\right)$	$1|G|+4|Z_{p}\left|+|M\right|$
Luo et al.	$2|Z_{p}|$	$\left(n+1\right)|G|+\left(n+2\right)|Z_{p}\left|+|M\right|$
Ours	$1|G|+2|Z_{p}|$	$5|G|+4|Z_{p}|$

*l*: the quantity of pseudo-identities generated for a vehicle in Xiong et al. [17]. *n*: the number of vehicles within the group.

#### 7.1.3. Comparison of Functions and Security

We compared our solution with those in [6,17,21] in Table 5, including functionality and security aspects. In the table, ‘⊥’ denotes aspects that are not discussed or proven in the respective schemes.

- Vehicle authentication is a crucial factor in conditional privacy-preserving schemes. The schemes in Table 5 authenticate vehicles either online or offline before distributing group keys.
- Our scheme achieves cross-domain and condition-matching for vehicles. These useful functionalities favor flexible vehicle management. Regrettably, other schemes do not consider these functionalities.
- To our knowledge, our scheme is the first in the literature pertaining to VANETs which supports conditional matching and cross-domain communication under conditional privacy preservation.
- Both our approach and the one presented in Xiong et al. [17] address dynamic scenarios involving the joining and leaving of vehicles in a group. In the event of changes in-group members, the group session key will be promptly updated to maintain the confidentiality of the group session following the changes. It is worth noting that certain alternative schemes lack provisions for managing dynamic member changes.
- In schemes [6,21], vehicle keys are generated offline by the TA and sent to vehicles using smart cards, incurring high usage costs. In Luo et al. [21], all keys are entrusted to the TA, posing insecurity if the TA is compromised. In Xiong et al. [17] and our scheme, certificateless cryptography is adopted, creating a key escrow-free scheme. Each vehicle generates a secret value and transmits the corresponding public information to the TA. The TA is responsible for generating a subset of keys, which, when combined with the secret value, forms the user’s key. Hence, the TA cannot access all elements of the vehicle keys. However, in Xiong et al. [17], pre-computed secret values are sent offline to vehicles, also incurring high usage costs.
- Both our proposed scheme and the one presented in Xiong et al. [17] have been demonstrated to attain forward secrecy and resist the attacks outlined in Table 5. It is noteworthy that not all other schemes exhibit these comprehensive security attributes.

### 7.2. Simulation

For the simulation of group sessions under conditional privacy protection, we used the Integer and Rational Arithmetic Cryptographic Library (Miracl) [41] to test the performance of our schemes and others, as presented in [6,17,21]. The experiments were performed on a desktop computer with a 64-bit Windows 10 operating system, featuring an Intel(R) Core(TM) i7-9700 CPU @ 3.00 GHz and 16.00 GB RAM.

We selected points belonging to the elliptic curve $E:\;y^{2}=x^{3}+x$ as elements of group *G*. The order of group *G* is denoted by *q*. The bit length of *q* is 256 bits, and the bit length of elements in *G* is 512 bits. We chose the eta_T pairing $e:G\times G\to G_{T}$ to evaluate the scheme [21]. The lengths of elements in *G*, $G_{T}$, and $Z_{p}$ are 512 bits, 512 bits, and 256 bits, respectively.

#### 7.2.1. Transmission Efficiency

**Figure 2:**
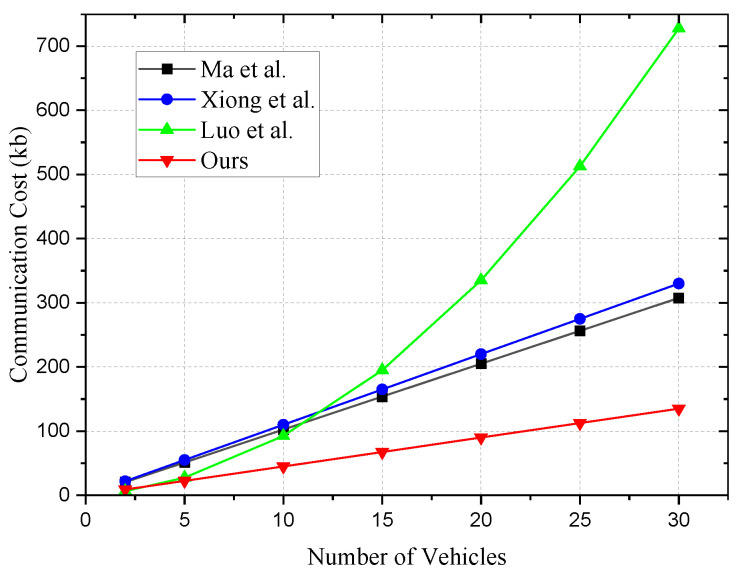
Transmission cost of vehicles [6,17,21].

The transmission costs for vehicles are shown in Table 6 and Figure 2. In our comparison, we consider the total transmission expenses for vehicles during both the registration and authentication processes. We set the group size *n* of vehicles to vary from 2, 5, 10 to 30. Below is the analysis of the transmission costs for vehicles:

- In Ma et al. [6], the authentication transmission cost when n=2 is 20.5 kb. As *n* increases from 5, 10 to 15, the transmission costs for vehicles are 51.25 kb, 102.5 kb, and 153.75 kb, respectively. When the number of vehicles reaches 30, the total transmission expense amounts to 307.5 kb.
- In Xiong et al. [17], the authentication transmission cost when n=2 is 22 kb. The communication volumes generated when *n* ranges from 5 to 30 are 55 kb, 110 kb, 165 kb, 220 kb, 275 kb, and 330 kb, respectively. The communication costs of this scheme are slightly higher compared to [6].
- In Luo et al. [21], due to the employment of a ring signature scheme, the transmission costs for each vehicle increase with the group size *n*. When n=2, the transmission cost for authenticating a single message is 6.5 kb. The communication costs generated when *n* ranges from 5 to 30 are 27.5 kb, 92.5 kb, 195 kb, 335 kb, 512.5 kb, and 727.5 kb, respectively. It is noticeable that this scheme incurs significantly higher communication costs as the number of vehicles increases compared to other schemes.
- In our scheme, when n=2, the authentication transmission cost is 9 kb. The communication costs generated when *n* ranges from 5 to 30 are 22.5 kb, 45 kb, 67.5 kb, 90 kb, 112.5 kb, and 135 kb, respectively. Our proposed scheme exhibits the most optimal communication costs.

**Table 6:** Transmission cost of vehicles (kb) [6,17,21].

*n*	2	5	10	15	20	25	30
Ma et al.	20.5	51.25	102.5	153.75	205	256.25	307.5
Xiong et al.	22	55	110	165	220	275	330
Luo et al.	6.5	27.5	92.5	195	335	512.5	727.5
Ours	9	22.5	45	67.5	90	112.5	135

In summary, compared to schemes in [6,17,21], our proposed scheme demonstrates lower communication transmission costs.

#### 7.2.2. Computation Efficiency

**Figure 3:**
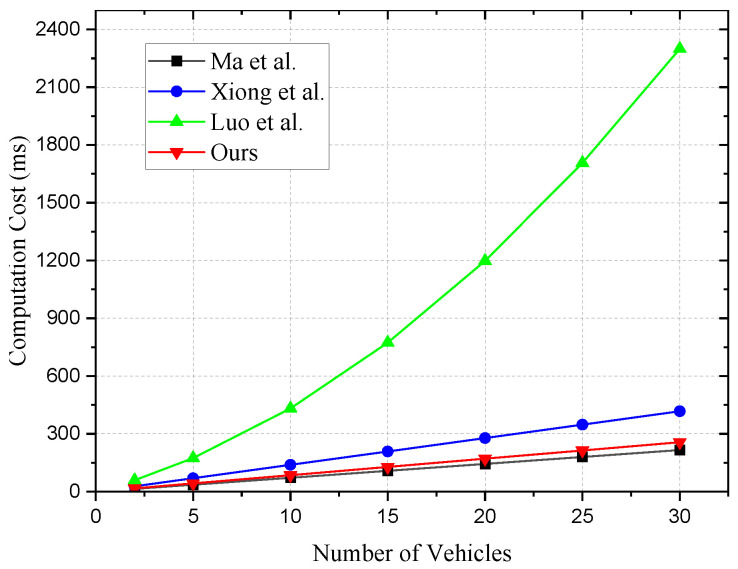
Computational cost of vehicles [6,17,21].

Next, we analyze computational efficiency. Bilinear pairing computations and hashing to points are particularly time-consuming, while scalar multiplications and hashing to $Z_{p}$ are more efficient operations. Especially, the addition computation in *G* is highly efficient, which we directly ignore in our analysis. It is essential to highlight that in Xiong et al. [17], the Chinese Remainder Theorem is used, and its construction and solving are also time-consuming computations that we must consider. Schemes [6,17], and our scheme use symmetric encryption for communication, which introduces encryption time considerations during communication. Table 7 and Figure 3 compare the computational costs for vehicles.

- In Ma et al. [6], the computation time for the authentication phase is $16T_{M}+18T_{H_{Z}}$. In our simulation test, the computation time for n=2 is 14.42 ms. As the communication quantity *n* increases from 5 to 30, the time increases from 36.05 ms to 216.3 ms. Hence, the computational time for the [6] scheme appears stable in Table 7.
- In Table 3, we analyzed the computational costs of each scheme with theory. In Xiong et al. [17], the computation costs for registration and authentication are $1T_{c}+1T_{r}+\left(2l+7\right)T_{M}+\left(l+4\right)T_{H_{Z}}$. The computation time for n=2 is 27.834 ms. As the number of vehicles *n* increases from 5 to 30, the time increases from 69.585 ms to 417.51 ms.
- In Luo et al. [21], the computation costs for vehicle registration and authentication processes are $3T_{P}+3nT_{PP}+\left(n+7\right)T_{pm}+1T_{H_{G}}+2T_{H_{Z}}$. The computation time for n=2 is 59.386 ms. This scheme employs a ring encryption method, hence the encryption algorithm’s computational load is substantial. As the number of vehicles *n* increases from 5 to 30, the time increases from 174.175 ms to 2299.05 ms.
- In our scheme, the computation cost for the authentication phase is $19T_{M}+15T_{H_{Z}}$. The computation time for n=2 is 17.11 ms. As the number of vehicles *n* increases from 5 to 30, the time increases from 42.775 ms to 256.65 ms.

Overall, in our proposed system, the computational costs for authentication and total communication remain at a lower level compared to all the compared schemes.

## 8. Conclusions

In this paper, we propose a dynamic privacy-preserving anonymous authentication scheme for condition-matching in fog-cloud-based VANETs. The approach addresses the challenge of computational limitations in OBUs by using general ECC to optimize computational efficiency. By leveraging fog computing, the scheme implements a multi-TA mode to enhance system robustness and meet the real-time requirements of VANETs. Our scheme employs a certificateless approach, eliminating the need for TA-managed certificates and enabling cross-domain group session key agreement. This improves the social aspects of VANETs and expands their potential applications in the era of intelligent vehicles. Integrating VANETs with cloud services enhances scalability and provides essential storage and computational support for diverse VANET-based applications. Our scheme satisfies the security requirements for conditional privacy protection in VANETs through security proofs. Additionally, performance analysis shows that it outperforms similar relevant schemes comprehensively. For future research, we consider designing authenticated key agreement based on lattices to achieve resistance against quantum attacks, and adopting outsourcing computing to reduce the computational requirements for vehicles.

## Figures and Tables

**Table 1:** Notations.

Notation	Description
*p*	a prime number
κ	security parameter
$a\in_{R}S$	*a* is randomly chosen from *S*
K	symmetric key space of SEnc/SDec
SEnc/SDec	secure symmetric encryption/decryption
$H_{0}$	secure hash function $H_{0}:{\left\{0,1\right\}}^{*}\to K$
$H_{i}$	secure hash function $H_{i}:{\left\{0,1\right\}}^{*}\to Z_{p}^{*}$ (1≤i≤6)
$\mathrm{TC}=(TC_{1},TC_{2},\cdots )$	a set of traffic conditions
TA	trusted authority
MPK/MSK	master public/secret key of the system
$FN_{\rho_{i}}$	the $\rho_{i}$-th fog node
$PID_{FN_{\rho_{i}}}$	the pseudo-identity of $FN_{\rho_{i}}$
$VH_{\rho_{i},\theta_{i}}$	the $\theta_{i}$-th vehicle in the $\rho_{i}$-th fog node domain
$PID_{VH_{\rho_{i},\theta_{i}}}$	the pseudo-identity of $VH_{\rho_{i},\theta_{i}}$
$PK_{FN_{\rho_{i}}}/SK_{FN_{\rho_{i}}}$	public/secret key of $FN_{\rho_{i}}$
$PK_{VH_{\rho_{i},\theta_{i}}}/SK_{VH_{\rho_{i},\theta_{i}}}$	public/secret key of $VH_{\rho_{i},\theta_{i}}$
$VT_{VH_{\rho_{i},\theta_{i}}}$	valid time of $VH_{\rho_{i},\theta_{i}}$’s public/secret keys
$VH_{\rho_{0},\theta_{0}}$	powerful vehicle
$\left\{VH_{\rho_{1},\theta_{1}},\cdot ,VH_{\rho_{n},\theta_{n}}\right\}$	low-power computation vehicles
GSK	group session key

**Table 2:** The notations of performance.

Notation	Description
$T_{HG}$	The average computation time for hash to *G*
$T_{HZ}$	The average computation time for hash to $Z_{p}$
$T_{M}$	The average computation time for scalar multiplication
$T_{PP}$	The computation time for exponentiation operations on the bilinear pairing $G_{T}$
$T_{P}$	The average computation time for bilinear pairing
$T_{cc}$	The computation time required to construct the Chinese Remainder Theorem
$T_{cr}$	The computation time for discovering the root of the Chinese Remainder Theorem
$|Z_{p}|$	The size of element in $Z_{p}$
|G|	The size of element in group *G*
$|G_{T}|$	The size of element in group $G_{T}$
|M|	The size of a typical message for vehicle communication.

**Table 5:** Comparison of functions and security [6,17,21].

Scheme	Ma et al.	Xiong et al.	Luo et al.	Ours
Authentication	√	√	√	√
Anonymity	√	√	√	√
Traceability	×	√	√	√
Cross-domain	×	×	×	√
Key Escrow-free	×	√	×	√
Condition-matching	×	×	×	√
Perfect forward secrecy	√	√	⊥	√
Resilience to replay attack	√	√	√	√
Resilience to impersonation attack	√	√	√	√
Resilience to modification attack	⊥	√	√	√

**Table 7:** Computational cost of vehicles (ms) [6,17,21].

*n*	2	5	10	15	20	25	30
Ma et al.	14.42	36.05	72.1	108.15	144.2	180.25	216.3
Xiong et al.	27.834	69.585	139.17	208.755	278.34	347.925	417.51
Luo et al.	59.386	174.175	431.95	773.325	1198.3	1706.875	2299.05
Ours	17.11	42.775	85.55	128.33	171.1	213.88	256.65

## Data Availability

Data are contained within the article.

## Supplementary Materials

The following supporting information can be downloaded at: https://www.mdpi.com/article/10.3390/s24061773/s1.
