# Supplementary material for: Dynamic Privacy-Preserving Anonymous Authentication Scheme for Condition-Matching in Fog-Cloud-Based VANETs
Source: Sensors (Basel). 2024 Mar 9;24(6):1773. doi: 10.3390/s24061773 (PMC10975316; doi:10.3390/s24061773)
Supplement: Supplementary file 1 [file sensors-24-01773-s001.zip › sensors-2861076-supplementary.pdf]

Supplemental Material

A. Correctness Analysis

The correctness of the proposed system is analyzed below.

Since $P_{pub} = xP$, $P_{FN_{\rho_i}} = x_{FN_{\rho_i}}P$, $R_{FN_{\rho_i}} = r_{FN_{\rho_i}}P$, $P_{VH_{\rho_i,\theta_i}} = x_{VH_{\rho_i,\theta_i}}P$, $R_{VH_{\rho_i,\theta_i}} = r_{VH_{\rho_i,\theta_i}}P$, $y_{FN_{\rho_i}} = \alpha_{FN_{\rho_i}}x + r_{FN_{\rho_i}}$, $y_{VH_{\rho_i,\theta_i}} = \beta_{VH_{\rho_i,\theta_i}}(x_{FN_{\rho_i}} + y_{FN_{\rho_i}}) + r_{VH_{\rho_i,\theta_i}}$ and $\Lambda_{VH_{\rho_i,\theta_i}} = a_{VH_{\rho_0,\theta_0}}y_{VH_{\rho_0,\theta_0}} \cdot P + [P_{VH_{\rho_i,\theta_i}} + \beta_{VH_{\rho_i,\theta_i}}(P_{FN_{\rho_i}} + \alpha_{FN_{\rho_i}}P_{pub} + R_{FN_{\rho_i}}) + R_{VH_{\rho_i,\theta_i}}]$, we can deduce that

$$\begin{aligned}
\Lambda'_{VH_{\rho_i,\theta_i}} &= \Lambda_{VH_{\rho_i,\theta_i}} - [a_{VH_{\rho_i,\theta_i}}y_{VH_{\rho_i,\theta_i}} \cdot P + (x_{VH_{\rho_i,\theta_i}} + y_{VH_{\rho_i,\theta_i}}) \cdot P] \\
&= a_{VH_{\rho_0,\theta_0}}y_{VH_{\rho_0,\theta_0}} \cdot P + [P_{VH_{\rho_i,\theta_i}} + \beta_{VH_{\rho_i,\theta_i}}(P_{FN_{\rho_i}} + \alpha_{FN_{\rho_i}}P_{pub} + R_{FN_{\rho_i}}) + \\
&\quad R_{VH_{\rho_i,\theta_i}}] - a_{VH_{\rho_i,\theta_i}}y_{VH_{\rho_i,\theta_i}} \cdot P - [x_{VH_{\rho_i,\theta_i}} + \\
&\quad \beta_{VH_{\rho_i,\theta_i}}(x_{FN_{\rho_i}} + \alpha_{FN_{\rho_i}}x + r_{FN_{\rho_i}}) + r_{VH_{\rho_i,\theta_i}}] \cdot P \\
&= (a_{VH_{\rho_0,\theta_0}}y_{VH_{\rho_0,\theta_0}} - a_{VH_{\rho_i,\theta_i}}y_{VH_{\rho_i,\theta_i}}) \cdot P.
\end{aligned}$$

Since

$$\Gamma_{VH_{\rho_i,\theta_i}} = a_{VH_{\rho_i,\theta_i}}y_{VH_{\rho_i,\theta_i}} \cdot P + [P_{VH_{\rho_0,\theta_0}} + \beta_{VH_{\rho_0,\theta_0}}(P_{FN_{\rho_0}} + \alpha_{FN_{\rho_0}}P_{pub} + R_{FN_{\rho_0}}) + R_{VH_{\rho_0,\theta_0}}],$$

we can deduce that

$$\begin{aligned}
\Gamma'_{VH_{\rho_i,\theta_i}} &= a_{VH_{\rho_0,\theta_0}}y_{VH_{\rho_0,\theta_0}} \cdot P + (x_{VH_{\rho_0,\theta_0}} + y_{VH_{\rho_0,\theta_0}}) \cdot P - \Gamma_{VH_{\rho_i,\theta_i}} \\
&= a_{VH_{\rho_0,\theta_0}}y_{VH_{\rho_0,\theta_0}} \cdot P + [x_{VH_{\rho_0,\theta_0}} + \\
&\quad \beta_{VH_{\rho_0,\theta_0}}(x_{FN_{\rho_0}} + \alpha_{FN_{\rho_0}}x + r_{FN_{\rho_0}}) + r_{VH_{\rho_0,\theta_0}}] \cdot P - \\
&\quad a_{VH_{\rho_i,\theta_i}}y_{VH_{\rho_i,\theta_i}} \cdot P - [P_{VH_{\rho_0,\theta_0}} + \\
&\quad \beta_{VH_{\rho_0,\theta_0}}(P_{FN_{\rho_0}} + \alpha_{FN_{\rho_0}}P_{pub} + R_{FN_{\rho_0}}) + R_{VH_{\rho_0,\theta_0}}] \\
&= (a_{VH_{\rho_0,\theta_0}}y_{VH_{\rho_0,\theta_0}} - a_{VH_{\rho_i,\theta_i}}y_{VH_{\rho_i,\theta_i}}) \cdot P.
\end{aligned}$$

Then, we have

$$\Lambda'_{VH_{\rho_i,\theta_i}} = \Gamma'_{VH_{\rho_i,\theta_i}} = (a_{VH_{\rho_0,\theta_0}}y_{VH_{\rho_0,\theta_0}} - a_{VH_{\rho_i,\theta_i}}y_{VH_{\rho_i,\theta_i}}) \cdot P.$$

$$\begin{aligned}
\Gamma_U &= \sum\nolimits_{VH_{\rho_i,\theta_i} \in U} \Gamma'_{VH_{\rho_i,\theta_i}} \\
&= \sum\nolimits_{VH_{\rho_i,\theta_i} \in U} a_{VH_{\rho_0,\theta_0}}a_{VH_{\rho_i,\theta_i}}y_{VH_{\rho_0,\theta_0}}y_{VH_{\rho_i,\theta_i}} \cdot P.
\end{aligned}$$

Since $A_{VH_{\rho_i,\theta_i}} = a_{VH_{\rho_i,\theta_i}} \cdot P$, $Z_{VH_{\rho_i,\theta_i}} = H_4(\Gamma_U) \cdot P + r_{VH_{\rho_0,\theta_0}}A_{VH_{\rho_i,\theta_i}}$ and $K_{VH_{\rho_0,\theta_0}} = H_4(\Gamma_U) \cdot P$, we can deduce that

$$\begin{aligned}
K_{VH_{\rho_i,\theta_i}} &= Z_{VH_{\rho_i,\theta_i}} - (a_{VH_{\rho_i,\theta_i}}) \cdot R_{VH_{\rho_0,\theta_0}} \\
&= H_4(\Gamma_U) \cdot P + r_{VH_{\rho_0,\theta_0}}A_{VH_{\rho_i,\theta_i}} - (a_{VH_{\rho_i,\theta_i}})R_{VH_{\rho_0,\theta_0}} \\
&= H_4(\Gamma_U) \cdot P \\
&= K_{VH_{\rho_0,\theta_0}}.
\end{aligned}$$

Then, we have

$$\begin{aligned}
Auth_{i,0} &= H_6(PID_U, \Lambda_{VH_{\rho_i,\theta_i}}, \Gamma_{VH_{\rho_i,\theta_i}}, \Lambda'_{VH_{\rho_i,\theta_i}}, K_{VH_{\rho_i,\theta_i}}) \\
&= H_6(PID_U, \Lambda_{VH_{\rho_i,\theta_i}}, \Gamma_{VH_{\rho_i,\theta_i}}, \Gamma'_{VH_{\rho_i,\theta_i}}, K_{VH_{\rho_0,\theta_0}}) \\
&= Auth_{0,i},
\end{aligned}$$

$$\begin{aligned}
GSK &= H_5(PID_U, PID_0, TC_{VH_{\rho_i,\theta_i}}, K_{VH_{\rho_i,\theta_i}}) \\
&= H_5(PID_U, PID_0, TC_{VH_{\rho_0,\theta_0}}, K_{VH_{\rho_0,\theta_0}}).
\end{aligned}$$

B. Security Proof

Theorem 1. *Under the random oracle model,The proposed system remains resilient to a $\mathcal{A}_{\mathcal{I}}$ type adversary provided that the decisional CDH assumption is upheld.*

***Proof*.** Let $\mathcal{A}$ represent a $\mathcal{A}_{\mathcal{I}}$ adversary targeting the system, capable of winning the ensuing interactive game with a non-negligible probability ϵ. A challenger $\mathcal{C}$ is devised with the capability to solve the CDH problem with a probability of success that is non-negligible.

Assigned with a random tuple $(P, Q_1 = aP, Q_2 = bP, T)$ representing the CDH problem, $\mathcal{C}$ is tasked with discerning whether $T = abP$ or T is chosen randomly from G.

Initialization $\mathcal{C}$ randomly designates a vehicle $VH_{I,J}$ as the challenged vehicle with lower computational power and another vehicle $VH_{0,0}$ as the challenged vehicle with relatively robust computation capabilities. $\mathcal{C}$ randomly selects $a_{VH_{I,J}}, a_{VH_{0,0}} \in_R Z_p^*$. Subsequently, $\mathcal{C}$ chooses $x \in_R Z_p^*$ and computes $P_{pub} = xP$. The master public key $MPK = (P, P_{pub})$ is then transmitted from $\mathcal{C}$ to $\mathcal{A}$. $\mathcal{C}$ responds to queries as outlined below.

- *Hash query:* $\mathcal{C}$ maintains an empty list H_i^{list} for each hash function H_i, where $1 \leq i \leq 6$. Upon receiving a hash query with message m_j on hash function H_i, $\mathcal{C}$ checks for the existence of a tuple (m_j, ν_j) in H_i^{list}. If the tuple is found, the value ν_i is returned. Otherwise, $\mathcal{C}$ selects a random $\nu_j \in_R Z_p^*$ and adds the tuple (m_j, ν_j) to H_i^{list}.
- *Symmetric encryption query:* The query results are stored in the list L_{SEnc}. Upon receiving a symmetric encryption query for m_i with key k_i, $\mathcal{C}$ checks for the existence of a tuple (m_i, k_i, c_i) in L_{SEnc}. If the tuple is found, the value c_i is returned. Otherwise, $\mathcal{C}$ selects a random $c_i \in_R Z_p^*$ and adds the tuple (m_i, k_i, c_i) to L_{SEnc}.
- *Extract secret value of FN_{ρ_i}*: The query results are stored in the list L_{FN}^1. When receiving a secret value extraction query for fog node FN_{ρ_i} with identity $ID_{FN_{\rho_i}}$, $\mathcal{C}$ checks for the existence of the tuple $(FN_{\rho_i}, ID_{FN_{\rho_i}}, P_{FN_{\rho_i}}, x_{FN_{\rho_i}})$ in L_{FN}^1. If found, the value $x_{FN_{\rho_i}}$ is returned. Otherwise, $\mathcal{C}$ selects a random $x_{FN_{\rho_i}} \in_R Z_p^*$ and computes $P_{FN_{\rho_i}} = x_{FN_{\rho_i}} P$. Then, the tuple $(FN_{\rho_i}, ID_{FN_{\rho_i}}, P_{FN_{\rho_i}}, x_{FN_{\rho_i}})$ is inserted into L_{FN}^1.
- *Extract partial secret key of FN_{ρ_i}*: The query results are maintained in the list L_{FN}^2. Upon receiving a partial secret key extraction query for fog node FN_{ρ_i}, $\mathcal{C}$ checks for the existence of the tuple $(FN_{\rho_i}, PID_{FN_{\rho_i}}, R_{FN_{\rho_i}}, y_{FN_{\rho_i}})$ in L_{FN}^2. If found, the value $x_{FN_{\rho_i}}$ is returned. Otherwise, $\mathcal{C}$ generates $(R_{FN_{\rho_i}}, y_{FN_{\rho_i}})$ following the scheme's protocol. Subsequently, the tuple $(FN_{\rho_i}, PID_{FN_{\rho_i}}, R_{FN_{\rho_i}}, y_{FN_{\rho_i}})$ is inserted into L_{FN}^2.
- *Request public key of FN_{ρ_i}*: The query results are stored in the list L_{FN}^3. Upon receiving a request for the public key of fog node FN_{ρ_i}, $\mathcal{C}$ checks for the existence of the tuple $(FN_{\rho_i}, P_{FN_{\rho_i}}, R_{FN_{\rho_i}})$ in L_{FN}^3. If found, the public key $(P_{FN_{\rho_i}}, R_{FN_{\rho_i}})$ is returned. Otherwise, $\mathcal{C}$ responds with $(P_{FN_{\rho_i}}, R_{FN_{\rho_i}})$ by accessing the L_{FN}^1 and L_{FN}^2 lists. Then, the tuple $(FN_{\rho_i}, P_{FN_{\rho_i}}, R_{FN_{\rho_i}})$ is inserted into L_{FN}^3.
- *Replace public key of FN_{ρ_i}*: The query results are stored in the list L_{FN}^4 in the form of a tuple $(FN_{\rho_i}, x_{FN_{\rho_i}}, P_{FN_{\rho_i}}, r_{FN_{\rho_i}}, R_{FN_{\rho_i}})$. Upon receiving a replace public key query with input $(FN_{\rho_i}, PK'_{FN_{\rho_i}})$, where $P'_{FN_{\rho_i}} = x'_{FN_{\rho_i}} P$, $R'_{FN_{\rho_i}} = r'_{FN_{\rho_i}} P$ and $PK'_{FN_{\rho_i}} = (P'_{FN_{\rho_i}}, R'_{FN_{\rho_i}})$, $\mathcal{C}$ inserts the tuple $(FN_{\rho_i}, x'_{FN_{\rho_i}}, P'_{FN_{\rho_i}}, r'_{FN_{\rho_i}}, R'_{FN_{\rho_i}})$ into L_{FN}^4.
- *Extract secret value of VH_{ρ_i,θ_i}*: The query results are stored in the list L_{VH}^1. When receiving a secret value extraction query for vehicle VH_{ρ_i,θ_i} with identity $ID_{VH_{\rho_i,\theta_i}}$, $\mathcal{C}$ checks for the existence of the tuple $(VH_{\rho_i,\theta_i}, ID_{VH_{\rho_i,\theta_i}}, P_{VH_{\rho_i,\theta_i}}, x_{VH_{\rho_i,\theta_i}})$ in L_{VH}^1. If found, the value $x_{VH_{\rho_i,\theta_i}}$ is returned. Otherwise, $\mathcal{C}$ selects $x_{VH_{\rho_i,\theta_i}} \in_R Z_p^*$ and computes $P_{VH_{\rho_i,\theta_i}} = x_{VH_{\rho_i,\theta_i}} P$. Then, the tuple $(VH_{\rho_i,\theta_i}, ID_{VH_{\rho_i,\theta_i}}, P_{VH_{\rho_i,\theta_i}}, x_{VH_{\rho_i,\theta_i}})$ is inserted into L_{VH}^1.
- *Extract partial secret key of VH_{ρ_i,θ_i}*: The query results are stored in the list L_{VH}^2. Obtaining a partial secret key extraction query on vehicle VH_{ρ_i,θ_i}, $\mathcal{C}$ checks whether $(VH_{\rho_i,\theta_i}, PID_{VH_{\rho_i,\theta_i}}, R_{VH_{\rho_i,\theta_i}}, y_{VH_{\rho_i,\theta_i}}, VT_{VH_{\rho_i,\theta_i}})$ exists in L_{VH}^2. If found, the value $y_{VH_{\rho_i,\theta_i}}$ is returned. Else, $\mathcal{C}$ sets $VT_{VH_{\rho_i,\theta_i}}, TC_{VH_{\rho_i,\theta_i}}$ as the valid time period and traffic condition of VH_{ρ_i,θ_i}, respectively. Then, $\mathcal{C}$ proceeds with the following calculations.
  - If $VH_{\rho_i,\theta_i} = VH_{I,J}$, $\mathcal{C}$ selects $\mu_{VH_{I,J}}, PID_{VH_{I,J}} \in_R Z_p^*$ and inserts the tuple $((ID_{VH_{I,J}}, \mu_{VH_{I,J}}), \perp, PID_{VH_{I,J}})$ into L_{SEnc}. Then, $\mathcal{C}$ selects $\beta_{VH_{I,J}} \in_R Z_p^*$, calculates $R_{VH_{I,J}} = Q_1 - a_{VH_{I,J}} \cdot P - \beta_{VH_{I,J}}(P_{FN_I} + \alpha_{FN_I} P_{pub} + R_{FN_I})$ and sets $y_{VH_{I,J}} = \perp$. After that, $\mathcal{C}$ inserts the tuple $(PID_{FN_I}, PID_{VH_{I,J}}, P_{VH_{I,J}}, R_{VH_{I,J}}, VT_{VH_{I,J}}, TC_{VH_{I,J}}, \beta_{VH_{I,J}})$ into H_2^{list}, and the tuple $(VH_{I,J}, PID_{VH_{I,J}}, R_{VH_{I,J}}, \perp, VT_{VH_{I,J}})$ into L_{VH}^2.
  - If $VH_{\rho_i,\theta_i} = VH_{0,0}$, $\mathcal{C}$ selects $\mu_{VH_{0,0}}, PID_{VH_{0,0}} \in_R Z_p^*$ and inserts the tuple $((ID_{VH_{0,0}}, \mu_{VH_{0,0}}), \perp, PID_{VH_{0,0}})$ into L_{SEnc}. Then, $\mathcal{C}$ selects $\beta_{VH_{0,0}} \in_R Z_p^*$ and calculates $R_{VH_{0,0}} = Q_2 - a_{VH_{0,0}} \cdot P - \beta_{VH_{0,0}}(P_{FN_0} + \alpha_{FN_0} P_{pub} + R_{FN_0})$ and sets $y_{VH_{0,0}} = \perp$. Then, $\mathcal{C}$ inserts the tuple $(PID_{FN_0}, PID_{VH_{0,0}}, P_{VH_{0,0}}, R_{VH_{0,0}}, VT_{VH_{0,0}}, TC_{VH_{0,0}}, \beta_{VH_{0,0}})$ into H_2^{list}, and the tuple $(VH_{0,0}, PID_{VH_{0,0}}, R_{VH_{0,0}}, \perp, VT_{VH_{0,0}})$ into L_{VH}^2.

- If $VH_{\rho_i,\theta_i} \neq VH_{I,J}, VH_{0,0}$, $\mathcal{C}$ generates $(R_{VH_{\rho_i,\theta_i}}, y_{VH_{\rho_i,\theta_i}})$ using the vehicle registration algorithm in the system. The tuple
$$(PID_{FN_{\rho_i}}, PID_{VH_{\rho_i,\theta_i}}, P_{VH_{\rho_i,\theta_i}}, R_{VH_{\rho_i,\theta_i}}, VT_{VH_{\rho_i,\theta_i}}, TC_{VH_{\rho_i,\theta_i}}, \beta_{VH_{\rho_i,\theta_i}})$$
is inserted into H_2^{list}, and the tuple
$$(VH_{\rho_i,\theta_i}, PID_{VH_{\rho_i,\theta_i}}, R_{VH_{\rho_i,\theta_i}}, y_{VH_{\rho_i,\theta_i}}, VT_{VH_{\rho_i,\theta_i}})$$
is inserted into L_{VH}^2.

- *Request public key of* VH_{ρ_i,θ_i}: The query result is maintained in the list L_{VH}^3. When a request for the public key query on vehicle VH_{ρ_i,θ_i} is received, $\mathcal{C}$ verifies the presence of the tuple $(VH_{\rho_i,\theta_i}, P_{VH_{\rho_i,\theta_i}}, R_{VH_{\rho_i,\theta_i}}, VT_{VH_{\rho_i,\theta_i}})$ in L_{VH}^3. If the tuple exists, $\mathcal{C}$ returns the associated public key $(P_{VH_{\rho_i,\theta_i}}, R_{VH_{\rho_i,\theta_i}}, VT_{VH_{\rho_i,\theta_i}})$. Otherwise, $\mathcal{C}$ responds $(P_{VH_{\rho_i,\theta_i}}, R_{VH_{\rho_i,\theta_i}}, VT_{VH_{\rho_i,\theta_i}})$ by accessing to the L_{VH}^1 and L_{VH}^2 lists. Then, the tuple $(VH_{\rho_i,\theta_i}, P_{VH_{\rho_i,\theta_i}}, R_{VH_{\rho_i,\theta_i}}, VT_{VH_{\rho_i,\theta_i}})$ is inserted to L_{VH}^3.
- *Replace public key of* VH_{ρ_i,θ_i}: The query results are stored in the list L_{VH}^4 in the form of a tuple
$$(VH_{\rho_i,\theta_i}, x_{VH_{\rho_i,\theta_i}}, P_{VH_{\rho_i,\theta_i}}, r_{VH_{\rho_i,\theta_i}}, R_{VH_{\rho_i,\theta_i}}, VT_{VH_{\rho_i,\theta_i}}).$$
When presented with a replace public key query containing the input $(VH_{\rho_i,\theta_i}, PK'_{VH_{\rho_i,\theta_i}})$, where
$$PK'_{VH_{\rho_i,\theta_i}} = (P'_{VH_{\rho_i,\theta_i}}, R'_{VH_{\rho_i,\theta_i}}, VT'_{VH_{\rho_i,\theta_i}}),$$
$$P'_{VH_{\rho_i,\theta_i}} = x'_{VH_{\rho_i,\theta_i}} P,\ R'_{VH_{\rho_i,\theta_i}} = r'_{VH_{\rho_i,\theta_i}} P,$$
$\mathcal{C}$ inserts the tuple $(VH_{\rho_i,\theta_i}, x'_{VH_{\rho_i,\theta_i}}, P'_{VH_{\rho_i,\theta_i}}, x'_{VH_{\rho_i,\theta_i}}, R'_{VH_{\rho_i,\theta_i}}, VT'_{VH_{\rho_i,\theta_i}})$ into L_{VH}^4.
- *Execute*: During the execution phase, the challenger $\mathcal{C}$ responds to the received message M.
  - $M =$ "Step 1": The query is the message $M =$ "Step 1", aiming to generate the Step 1 message from VH_{ρ_i,θ_i} to VH_{ρ_j,θ_j}.
    - ◇ If $VH_{\rho_i,\theta_i} = VH_{I,J}$, $\mathcal{C}$ terminates the game.
    - ◇ If $VH_{\rho_i,\theta_i} \neq VH_{I,J}$ and $VH_{\rho_j,\theta_j} = VH_{0,0}$, $\mathcal{C}$ terminates the game.
    - ◇ If $VH_{\rho_i,\theta_i} \neq VH_{I,J}$ and $VH_{\rho_j,\theta_j} \neq VH_{0,0}$, the challenger $\mathcal{C}$ generates $(A_{VH_{\rho_i,\theta_i}}, b_{VH_{\rho_i,\theta_i}}, \Gamma_{VH_{\rho_i,\theta_i}})$ following the scheme protocol.
  - $M =$ "Step 2": The query is the message $M =$ "Step 2", intended to generate the Step 2 message from VH_{ρ_i,θ_i} to VH_{ρ_j,θ_j}.
    - ◇ If $VH_{\rho_i,\theta_i} = VH_{0,0}$, $\mathcal{C}$ terminates the game.
    - ◇ If $VH_{\rho_i,\theta_i} \neq VH_{0,0}$ and $VH_{\rho_j,\theta_j} = VH_{I,J}$, $\mathcal{C}$ terminates the game.
    - ◇ If $VH_{\rho_i,\theta_i} \neq VH_{0,0}$ and $VH_{\rho_j,\theta_j} \neq VH_{I,J}$, $\mathcal{C}$ generates $(Auth_{0,i}, PID_U, Z_{VH_{\rho_i,\theta_i}}, \Lambda_{VH_{\rho_i,\theta_i}})$ based on the scheme protocol.
  - $M =$ "Step 3": The query is the message $M =$ "Step 3", and $\mathcal{C}$ performs the actions outlined in Step 3 of the scheme protocol.

- *Reveal group session key*: When processing the group session key request, $\mathcal{C}$ verifies if the group member possesses neither $VH_{I,J}$ nor $VH_{0,0}$. If either is found, $\mathcal{C}$ halts the process. Otherwise, $\mathcal{C}$ proceeds to generate the group session key using the key agreement protocol specified in the CD-AGKMS system.
- *Corrupt* FN_{ρ_i}: Obtaining the corrupt query on fog node FN_{ρ_i}, $\mathcal{C}$ looks up L_{FN}^1 and L_{FN}^2 for the tuples $(FN_{\rho_i}, ID_{FN_{\rho_i}}, P_{FN_{\rho_i}}, x_{FN_{\rho_i}})$ and $(FN_{\rho_i}, PID_{FN_{\rho_i}}, R_{FN_{\rho_i}}, y_{FN_{\rho_i}})$. Then, $\mathcal{C}$ returns to $\mathcal{A}$ the tuple $(P_{FN_{\rho_i}}, R_{FN_{\rho_i}}, x_{FN_{\rho_i}}, y_{FN_{\rho_i}})$.
- *Corrupt* VH_{ρ_i,θ_i}: Obtaining the corrupt query on vehicle VH_{ρ_i,θ_i}, $\mathcal{C}$ looks up L_{VH}^1 and L_{VH}^2 for the tuples $(VH_{\rho_i,\theta_i}, ID_{VH_{\rho_i,\theta_i}}, P_{VH_{\rho_i,\theta_i}}, x_{VH_{\rho_i,\theta_i}})$ and $(VH_{\rho_i,\theta_i}, PID_{VH_{\rho_i,\theta_i}}, R_{VH_{\rho_i,\theta_i}}, y_{VH_{\rho_i,\theta_i}}, VT_{VH_{\rho_i,\theta_i}})$. Then, $\mathcal{C}$ returns $(P_{VH_{\rho_i,\theta_i}}, R_{VH_{\rho_i,\theta_i}}, VT_{VH_{\rho_i,\theta_i}}, x_{VH_{\rho_i,\theta_i}}, y_{VH_{\rho_i,\theta_i}})$ to $\mathcal{A}$.
- *Testing Phase*: During this stage, $\mathcal{C}$ randomly chooses $b \in_R \{0, 1\}$.
  - If $b = 1$, $\mathcal{C}$ generates authentication details for the interaction involving the challenge vehicles $VH_{0,0}$ and $VH_{I,J}$. Specifically, $\mathcal{C}$ calculates $\Gamma'_{VH_{I,J}} = T$, $\Gamma_U = \sum_{VH_{\rho_i,\theta_i} \in U} \Gamma'_{VH_{\rho_i,\theta_i}}$, $K_{VH_{0,0}} = H_4(\Gamma_U) \cdot P$, $Z_{VH_{I,J}} = H_4(\Gamma_U) \cdot P + a_{VH_{I,J}} R_{VH_{0,0}}$, $\Lambda_{VH_{I,J}} = x_{VH_{I,J}} Q_1 + T$, and $Auth_{0,i} = H_6(PID_U, \Lambda_{VH_{\rho_i,\theta_i}}, \Gamma_{VH_{\rho_i,\theta_i}}, \Gamma'_{VH_{\rho_i,\theta_i}}, K_{VH_{\rho_0,\theta_0}})$. Afterwards, $\mathcal{C}$ transmits the tuple $(Auth_{0,i}, PID_U, Z_{VH_{I,J}}, \Lambda_{VH_{I,J}})$ back to $\mathcal{A}$.
  - If $b = 0$, $\mathcal{C}$ randomly selects $(Auth_{0,i}, Z_{VH_{I,J}}, \Lambda_{VH_{I,J}})$ and conveys the randomly chosen authentication information $(Auth_{0,i}, PID_U, Z_{VH_{I,J}}, \Lambda_{VH_{I,J}})$ to $\mathcal{A}$.

Finally, $\mathcal{A}$ produces an outcome denoted as $b' \in \{0, 1\}$. If b' matches b, $\mathcal{A}$ emerges victorious in the game. Following this, $\mathcal{C}$ could solve the CDH problem by distinguishing whether $T = abP$ or if T represents a random element.

C. Security Requirements Analysis

Theorem 3. *The proposed system satisfies mutual authentication, fog node anonymity, vehicle anonymity, group key establishment, vehicle traceability, management of authenticated keys across different domains , condition-matching, time controlled revocation, perfect forward secrecy, impersonation/modification/replay attack resistance.*

In this context, we demonstrate that the proposed system fulfills the system criteria outlined in Section 3.2.

C.1. Mutual authentication

In the group key agreement phase, each vehicle $VH_{\rho_i,\theta_i} \in U_0$ sends the tuple $(A_{VH_{\rho_i,\theta_i}}, b_{VH_{\rho_i,\theta_i}}, \Gamma_{VH_{\rho_i,\theta_i}})$ to VH_{ρ_0,θ_0}. Receiving the message, VH_{ρ_0,θ_0} verifies whether the equation holds:

$$\begin{aligned} & b_{VH_{\rho_i,\theta_i}} - (A_{VH_{\rho_i,\theta_i}} + \gamma_{VH_{\rho_i,\theta_i}} P) \\ = \ & P_{VH_{\rho_i,\theta_i}} + \beta_{VH_{\rho_i,\theta_i}} (P_{FN_{\rho_i}} + \alpha_{FN_{\rho_i}} P_{pub} + R_{FN_{\rho_i}}) + R_{VH_{\rho_i,\theta_i}}. \end{aligned}$$

As the legitimate $b_{VH_{\rho_i,\theta_i}}$ is computable exclusively through the vehicle's confidential key $SK_{VH_{\rho_i,\theta_i}} = (x_{VH_{\rho_i,\theta_i}}, y_{VH_{\rho_i,\theta_i}})$, the identity of the vehicle VH_{ρ_i,θ_i} can be authenticated by VH_{ρ_0,θ_0}.

In step 2, the powerful vehicle VH_{ρ_0,θ_0} calculates $\Gamma'_{VH_{\rho_i,\theta_i}}$ using its own secret key $SK_{VH_{\rho_0,\theta_0}} = (x_{VH_{\rho_0,\theta_0}}, y_{VH_{\rho_0,\theta_0}})$. In step 3, the vehicle VH_{ρ_i,θ_i} calculates

$$\Lambda'_{VH_{\rho_i,\theta_i}} = \Lambda_{VH_{\rho_i,\theta_i}} - [a_{VH_{\rho_i,\theta_i}} y_{VH_{\rho_i,\theta_i}} \cdot P + (x_{VH_{\rho_i,\theta_i}} + y_{VH_{\rho_i,\theta_i}}) \cdot P].$$

If the $SK_{VH_{\rho_0,\theta_0}}$ for VH_{ρ_0,θ_0} is deceptive, the equation $\Gamma''_{VH_{\rho_i,\theta_i}} = \Lambda''_{VH_{\rho_i,\theta_i}}$ doesn't hold. Consequently, the vehicle VH_{ρ_i,θ_i} detects the falsified information, given that $Auth_{i,0} = Auth_{0,i}$. As a result, the vehicle with superior computational capability is successfully authenticated.

Within this system, a high-powered vehicle is tasked with authenticating each low-power computation-capable vehicle. Simultaneously, the high-powered vehicle is subject to authentication by every low-power computation-capable vehicle. (The low-power computation-capable vehicles do not authenticate each other due to the absence of communication.) As a result, our system achieves group vehicle authentication based on condition matching.

C.2. Fog node anonymity

In accordance with Subsection 4.2, the identity $ID_{FN_{\rho_i}}$ of the fog node is encrypted using a symmetric encryption algorithm with the master secret key. Its pseudonymous identity $PID_{FN_{\rho_i}}$ is computed as

$$PID_{FN_{\rho_i}} = SEnc_{H_0(x)}(ID_{FN_{\rho_i}}, \mu_{FN_{\rho_i}}).$$

The attacker cannot recover $ID_{FN_{\rho_i}}$ from $PID_{FN_{\rho_i}}$ due to the cryptographic security of the $SEnc$ algorithm, and the attacker is unable to obtain the master secret key $MSK = x$. As a result, our system ensures fog node anonymity.

C.3. Vehicle anonymity

During the vehicle registration process outlined in Subsection 4.3, the identity $ID_{VH_{\rho_i,\theta_i}}$ of the vehicle undergoes a transformation into the pseudonymous identity $PID_{VH_{\rho_i,\theta_i}}$ by the fog node FN_{ρ_i} using its confidential key $SK_{FN_{\rho_i}} = (x_{FN_{\rho_i}}, y_{FN_{\rho_i}})$. The computation of the vehicle's pseudonymous identity is expressed as

$$PID_{VH_{\rho_i,\theta_i}} = SEnc_{H_0(x_{FN_{\rho_i}}, y_{FN_{\rho_i}})}(ID_{VH_{\rho_i,\theta_i}}, \mu_{VH_{\rho_i,\theta_i}}).$$

As $SK_{FN_{\rho_i}}$ is securely stored by FN_{ρ_i} and the security of the symmetric encryption algorithm is assured, the attacker is incapable of deducing the actual vehicle identity $ID_{VH_{\rho_i,\theta_i}}$ from $PID_{VH_{\rho_i,\theta_i}}$. Consequently, our scheme guarantees vehicle anonymity.

C.4. Fog node traceability

When a fog node FN_{ρ_i} behaves maliciously, the TA can recover its real identity $ID_{FN_{\rho_i}}$ by decrypting $PID_{FN_{\rho_i}}$ using the master secret key x:

$$(ID_{FN_{\rho_i}}, \mu_{FN_{\rho_i}}) = SDec_{H_0(x)}(PID_{FN_{\rho_i}}).$$

Thus, this proposed system provides fog node traceability.

C.5. Vehicle traceability

If a vehicle VH_{ρ_i,θ_i} spreads rumours in the group chat room, the fog node FN_{ρ_i} can find his real identity $ID_{VH_{\rho_i,\theta_i}}$ using the secret key $SK_{FN_{\rho_i}} = (x_{FN_{\rho_i}}, y_{FN_{\rho_i}})$:

$$(ID_{VH_{\rho_i,\theta_i}}, \mu_{VH_{\rho_i,\theta_i}}) = SDec_{H_0(x_{FN_{\rho_i}}, y_{FN_{\rho_i}})}(PID_{VH_{\rho_i,\theta_i}}).$$

Then, the vehicle traceability is achieved in this system.

C.6. Session key establishment

Built upon the correctness analysis, both the formidable vehicle VH_{ρ_0,θ_0} and the vehicle with low-power computation capability VH_{ρ_i,θ_i} $(1 \le i \le n)$ can collectively compute the group session key:

$$\begin{aligned} GSK &= H_1(PID_U, PID_0, TC_{VH_{\rho_i,\theta_i}}, K_{VH_{\rho_i,\theta_i}}) \\ &= H_1(PID_U, PID_0, TC_{VH_{\rho_0,\theta_0}}, K_{VH_{\rho_0,\theta_0}}). \end{aligned}$$

Subsequently, the achievement of group session key agreement is demonstrated in this system.

C.7. Cross-domain authenticated key agreement

In our proposed approach, vehicles VH_{ρ_0,θ_0} and VH_{ρ_i,θ_i} $(1 \le i \le n)$ individually complete registration processes with the respective fog nodes FN_{ρ_0} and FN_{ρ_i} $(1 \le i \le n)$. Through a rigorous authentication process, a group key is established. This design effectively achieves authenticated key management across different domains.

C.8. Traffic condition matching

In our proposed scheme, the secret key of a vehicle, denoted as $y_{VH_{\rho_i,\theta_i}}$, is dynamically influenced by the inclusion of traffic conditions, represented by $TC_{VH_{\rho_i,\theta_i}}$. This dynamic key formulation is expressed as $y_{VH_{\rho_i,\theta_i}} = \beta_{VH_{\rho_i,\theta_i}}(x_{FN_{\rho_i}} + y_{FN_{\rho_i}}) + r_{VH_{\rho_i,\theta_i}} \mod p$, where $\beta_{VH_{\rho_i,\theta_i}} = H_1(PID_{FN_{\rho_i}}, PID_{VH_{\rho_i,\theta_i}}, P_{VH_{\rho_i,\theta_i}}, R_{VH_{\rho_i,\theta_i}}, VT_{VH_{\rho_i,\theta_i}}, TC_{VH_{\rho_i,\theta_i}})$.

During the group key agreement phase, the vehicle VH_{ρ_i,θ_i} transmits $b_{VH_{\rho_i,\theta_i}} = (a_{VH_{\rho_i,\theta_i}} + \gamma_{VH_{\rho_i,\theta_i}} + x_{VH_{\rho_i,\theta_i}} + y_{VH_{\rho_i,\theta_i}}) \cdot P$ to VH_{ρ_0,θ_0}.

VH_{ρ_0,θ_0} then validates whether

$$\begin{aligned} & b_{VH_{\rho_i,\theta_i}} - (A_{VH_{\rho_i,\theta_i}} + \gamma_{VH_{\rho_i,\theta_i}} P) \\ = \; & P_{VH_{\rho_i,\theta_i}} + \beta_{VH_{\rho_i,\theta_i}}(P_{FN_{\rho_i}} + \alpha_{FN_{\rho_i}} P_{pub} + R_{FN_{\rho_i}}) + R_{VH_{\rho_i,\theta_i}}. \end{aligned}$$

where

$$\begin{aligned} \alpha_{FN_{\rho_i}} &= H_1(PID_{FN_{\rho_i}}, P_{FN_{\rho_i}}, R_{FN_{\rho_i}}), \\ \beta_{VH_{\rho_i,\theta_i}} &= H_2(PID_{FN_{\rho_i}}, PID_{VH_{\rho_i,\theta_i}}, P_{VH_{\rho_i,\theta_i}}, R_{VH_{\rho_i,\theta_i}}, VT_{VH_{\rho_i,\theta_i}}, TC_{VH_{\rho_0,\theta_0}}), \\ \gamma_{VH_{\rho_i,\theta_i}} &= H_3(A_{VH_{\rho_i,\theta_i}}, P_{VH_{\rho_i,\theta_i}}, R_{VH_{\rho_i,\theta_i}}, VT_{VH_{\rho_i,\theta_i}}, TC_{VH_{\rho_0,\theta_0}}). \end{aligned}$$

If the group vehicles VH_{ρ_0,θ_0} and VH_{ρ_i,θ_i} $(1 \le i \le n)$ exhibit disparate traffic conditions, a successful authentication is unattainable. Additionally, the establishment of the condition-matching-based group session key, denoted as GSK, faces hindrance, as represented by

$$GSK = H_1(PID_U, PID_0, TC_{VH_{\rho_i,\theta_i}}, K_{VH_{\rho_i,\theta_i}}).$$

Hence, the realization of the condition-matching function is exemplified within this system.

C.9. Time-limited keys

Within our system, the valid time period $VT_{VH_{\rho_i,\theta_i}}$ is explicitly integrated into the calculation of the vehicle's secret key:

$$y_{VH_{\rho_i,\theta_i}} = \beta_{VH_{\rho_i,\theta_i}}(x_{FN_{\rho_i}} + y_{FN_{\rho_i}}) + r_{VH_{\rho_i,\theta_i}},$$

where

$$\beta_{VH_{\rho_i,\theta_i}} = H_1(PID_{FN_{\rho_i}}, PID_{VH_{\rho_i,\theta_i}}, P_{VH_{\rho_i,\theta_i}}, R_{VH_{\rho_i,\theta_i}}, VT_{VH_{\rho_i,\theta_i}}, TC_{VH_{\rho_i,\theta_i}}).$$

In the group key agreement phase, the vehicle VH_{ρ_i,θ_i} submits $b_{VH_{\rho_i,\theta_i}} = (a_{VH_{\rho_i,\theta_i}} + \gamma_{VH_{\rho_i,\theta_i}} + x_{VH_{\rho_i,\theta_i}} + y_{VH_{\rho_i,\theta_i}}) \cdot P$ to VH_{ρ_0,θ_0}.

VH_{ρ_0,θ_0} verifies whether

$$\begin{aligned} & b_{VH_{\rho_i,\theta_i}} - (A_{VH_{\rho_i,\theta_i}} + \gamma_{VH_{\rho_i,\theta_i}} P) \\ = \; & P_{VH_{\rho_i,\theta_i}} + \beta_{VH_{\rho_i,\theta_i}}(P_{FN_{\rho_i}} + \alpha_{FN_{\rho_i}} P_{pub} + R_{FN_{\rho_i}}) + R_{VH_{\rho_i,\theta_i}}. \end{aligned}$$

where

$$\begin{aligned} \alpha_{FN_{\rho_i}} &= H_1(PID_{FN_{\rho_i}}, P_{FN_{\rho_i}}, R_{FN_{\rho_i}}), \\ \beta_{VH_{\rho_i,\theta_i}} &= H_2(PID_{FN_{\rho_i}}, PID_{VH_{\rho_i,\theta_i}}, P_{VH_{\rho_i,\theta_i}}, R_{VH_{\rho_i,\theta_i}}, VT_{VH_{\rho_i,\theta_i}}, TC_{VH_{\rho_0,\theta_0}}), \\ \gamma_{VH_{\rho_i,\theta_i}} &= H_3(A_{VH_{\rho_i,\theta_i}}, P_{VH_{\rho_i,\theta_i}}, R_{VH_{\rho_i,\theta_i}}, VT_{VH_{\rho_i,\theta_i}}, TC_{VH_{\rho_0,\theta_0}}). \end{aligned}$$

Should the valid time period $VT_{VH_{\rho_i,\theta_i}}$ elapse, successful authentication becomes unattainable.

Consequently, the implementation of time-controlled vehicle revocation is actualized within this system.

C.10. Perfect forward secrecy

In the scenario where an adversary successfully acquires the secret keys of vehicles VH_{ρ_0,θ_0} and VH_{ρ_i,θ_i} $(1 \leq i \leq n)$ and intercepts the transmitted messages $(A_{VH_{\rho_i,\theta_i}}, b_{VH_{\rho_i,\theta_i}}, \Gamma_{VH_{\rho_i,\theta_i}})$ and $(Auth_{0,i}, PID_U, Z_{VH_{\rho_i,\theta_i}}, \Lambda_{VH_{\rho_i,\theta_i}})$.

Utilizing the obtained secret keys, the attacker calculates:

$$\begin{aligned} & \Lambda_{VH_{\rho_i,\theta_i}} - [y_{VH_{\rho_i,\theta_i}} \cdot P + (x_{VH_{\rho_i,\theta_i}} + y_{VH_{\rho_i,\theta_i}}) \cdot P] \\ = \; & a_{VH_{\rho_0,\theta_0}} y_{VH_{\rho_0,\theta_0}} \cdot P + [P_{VH_{\rho_i,\theta_i}} + \beta_{VH_{\rho_i,\theta_i}} (P_{FN_{\rho_i}} + \alpha_{FN_{\rho_i}} P_{pub} + R_{FN_{\rho_i}}) + \\ & R_{VH_{\rho_i,\theta_i}}] - y_{VH_{\rho_i,\theta_i}} \cdot P - [x_{VH_{\rho_i,\theta_i}} + \\ & \beta_{VH_{\rho_i,\theta_i}} (x_{FN_{\rho_i}} + \alpha_{FN_{\rho_i}} x + r_{FN_{\rho_i}}) + r_{VH_{\rho_i,\theta_i}}] \cdot P \\ = \; & (a_{VH_{\rho_0,\theta_0}} y_{VH_{\rho_0,\theta_0}} - y_{VH_{\rho_i,\theta_i}}) \cdot P. \end{aligned}$$

If the attacker cannot solve the DL problem, they are unable to derive $a_{VH_{\rho_i,\theta_i}}$ from $A_{VH_{\rho_i,\theta_i}} = a_{VH_{\rho_i,\theta_i}} P$.

Thus, the attacker cannot deduce $K_{VH_{\rho_i,\theta_i}} = Z_{VH_{\rho_i,\theta_i}} - (a_{VH_{\rho_i,\theta_i}}) \cdot R_{VH_{\rho_0,\theta_0}}$ or $\Lambda'_{VH_{\rho_i,\theta_i}} = (a_{VH_{\rho_0,\theta_0}} y_{VH_{\rho_0,\theta_0}} - a_{VH_{\rho_i,\theta_i}} y_{VH_{\rho_i,\theta_i}}) \cdot P$.

Without traffic condition information and $K_{VH_{\rho_i,\theta_i}}$, the attacker is unable to compute the group session key $GSK = H_1(PID_U, PID_0, TC_{VH_{\rho_i,\theta_i}}, K_{VH_{\rho_i,\theta_i}})$.

Hence, the inherent complexity of the DL problem ensures the perfect forward secrecy of this cryptographic scheme.

C.11. Resist replay attack

In the group key agreement protocol, both vehicles VH_{ρ_0,θ_0} and VH_{ρ_i,θ_i} $(1 \leq i \leq n)$ generate new random numbers, namely $a_{VH_{\rho_0,\theta_0}} \in Z_p^*$ and $a_{VH_{\rho_i,\theta_i}} \in Z_p^*$, respectively. These randomly chosen numbers play a crucial role in the authentication information $Auth_{i,0}$ and $Auth_{0,i}$, as indicated by the relationship:

$$\Lambda'_{VH_{\rho_i,\theta_i}} = \Gamma'_{VH_{\rho_i,\theta_i}} = (a_{VH_{\rho_0,\theta_0}} y_{VH_{\rho_0,\theta_0}} - a_{VH_{\rho_i,\theta_i}} y_{VH_{\rho_i,\theta_i}}) \cdot P.$$

Given that these values $a_{VH_{\rho_0,\theta_0}}, b_{VH_{\rho_0,\theta_0}}, a_{VH_{\rho_i,\theta_i}}, b_{VH_{\rho_i,\theta_i}} \in Z_p^*$ are chosen randomly for each group key agreement, any attempt by an attacker to replay eavesdropped messages will be promptly detected by the vehicles through the verification of the authentication information. Consequently, our system effectively withstands replay attacks.

C.12. Resist impersonation attack

In Section 3.3 we introduce $\mathcal{A_I}$ and $\mathcal{A_{II}}$ adversaries, representing external and internal attackers, respectively. The security model is explicitly defined in Section 3.3. The interactive game serves to emulate the interaction between the vehicle and the adversary.

Drawing upon Theorems 1 to 2, it becomes evident that no polynomial-time attacker possesses the capability to forge the interactive information of the vehicles. Consequently, the group members can identify impersonation attacks by scrutinizing the received messages. Our system demonstrates robust security against impersonation attacks.

C.13. Resist tampering attack

During the third step of the group key agreement phase, the authentication information $Auth_{i,0}$ and $Auth_{0,i}$ are exchanged between VH_{ρ_0,θ_0} and VH_{ρ_i,θ_i} $(1 \leq i \leq n)$. These values are computed under the key $K_{VH_{\rho_0,\theta_0}} = K_{VH_{\rho_i,\theta_i}} = \delta_{VH_{\rho_0,\theta_0}} \cdot P$. Importantly, $K_{VH_{\rho_0,\theta_0}}$ is securely retained by VH_{ρ_0,θ_0} and is not transmitted during communication.

The key $K_{VH_{\rho_i,\theta_i}}$ is computed by VH_{ρ_i,θ_i} using their secret key $SK_{VH_{\rho_i,\theta_i}}$ and the random numbers $a_{VH_{\rho_i,\theta_i}}, b_{VH_{\rho_i,\theta_i}} \in Z_p^*$. Consequently, the attacker is unable to deduce the key $K_{VH_{\rho_i,\theta_i}}$, preventing them from generating valid authentication information in the event of communication content modification.

Any attempt to modify the authentication information using an invalid key can be detected by checking the equation $Auth_{i,0} = Auth_{0,i}$. Therefore, our system exhibits resilience against modification attacks.
